# Oxytocin receptor signaling in the dorsal lateral septum is essential for social transmission of food preference in mice

**DOI:** 10.1371/journal.pbio.3004044

**Published:** 2026-10-14

**Authors:** Hanyu Fu, Zhiqiu Liu, Jingwei Zhou, Luyao Zhang, Cai Liu, Dewei Tang, Shuangshuang Zhang, Anan Li, Dejuan Wang

**Affiliations:** 1 Jiangsu Key Laboratory of Brain Disease Bioinformation, Research Center for Biochemistry and Molecular Biology, Xuzhou Medical University, Xuzhou, Jiangsu, China; 2 Schools of Basic Medicine and Life Science, Xuzhou Medical University, Xuzhou, Jiangsu, China; INSERM, FRANCE

## Abstract

Social learning is a fundamental adaptive strategy that critically shapes decision-making and feeding behaviors. Through the social transmission of food preference (STFP), animals acquire information about food safety via social interactions, forming a food-odor memory that overrides innate preferences and guides subsequent food preferences. However, the neural mechanisms through which social cues durably influence feeding preferences remain unclear. Here, we identify the lateral septum (LS) as a key node in STFP. We show that LS neuronal activity increases following social learning and reliably distinguishes between socially cued and uncued foods. Chemogenetic inhibition of the dorsal LS (dLS), but not the ventral LS (vLS), abolishes STFP-driven food preference, revealing subregional specificity within the LS. Furthermore, oxytocin receptor (OXTR) signaling within the dLS is required for STFP in mice, where it enhances neuronal excitability through PLC pathway activation. Together, our results reveal a subregion-specific circuit mechanism through which the dLS integrates social information with feeding motivation, and highlight oxytocinergic modulation as a key mediator of socially guided food preferences.

## Abstract 2

Social learning is a fundamental adaptive strategy that shapes decision-making and feeding behaviors in animals via the social transmission of food preference (STFP), but the mechanisms underlying this process remain unclear. This study shows that oxytocin signaling in the dorsal lateral septum is required for SFTP in mice.

## Introduction

Social learning represents a core adaptive strategy in both animals and humans, critically modulating decision-making and feeding behaviors. The social transmission of food preference (STFP) enables individuals to acquire information about food safety between social conspecifics, generating a food-odor memory that overrides innate food preferences and bridges social experience with food choice [1–3]. Yet, the precise neural biological mechanisms through which social cues durably influence feeding preferences remain poorly understood.

In rodents, the formation of STFP relies fundamentally on olfactory-guided social interaction [4,5]. During STFP, socially derived food odors are detected and processed by dedicated olfactory centers including the olfactory bulb, anterior olfactory nucleus, and the piriform cortex [2,3,6]. The social and sensory olfactory inputs are then integrated and converted into a stable social memory in brain regions including the orbitofrontal cortex, medial prefrontal cortex, and ventral hippocampus, and is subsequently consolidated for long-term storage in the cortical amygdala [1,7,8]. The hippocampus and its interconnected pathways are critically involved in processing social information, particularly in social learning [5,7,9–11].

The lateral septum (LS) serves as an important downstream region of the hippocampus and is characterized by dense reciprocal projections between the two structures [12-14]. Activation of the LS inputs to hippocampus facilitate social learning [12], whereas LS efferents relay contextual information from the hippocampus to midbrain and hypothalamic regions, allowing the LS to adaptively integrate environmental cues with motivational state and refine feeding behavior [14]. These findings suggest that the LS may act as an integrative adaptor that translates social memory into feeding responses via downstream motivational circuits. Notably, local LS circuits exert fine-tuned control on feeding through pathway-specific outputs: projections to tuberal nucleus selectively suppress hedonic feeding, while outputs to the supramammillary nucleus contributes to broader satiety signaling and anorectic effect [15,16]. Therefore, the LS may act as a critical node for modulating STFP, implementing a putative neural mechanism that integrates social memory signals with internal feeding motivation to guide food-choice behavior. Importantly, LS also expresses high levels of oxytocin and vasopressin receptors, neuropeptide systems that modulate social behavior in a sex- and social context-dependent ways [17,18]. However, the functional role of these neuropeptide systems within the LS in mediating STFP is unknown.

In this study, we first characterize LS neural dynamics during STFP via real-time monitoring of calcium transients and spike activity. We then defined the necessity of the dorsal LS (dLS) and ventral (vLS) in STFP by precise neuronal manipulation. Finally, we examined the contribution of oxytocin receptor (OXTR) signaling in LS to STFP behavior, aiming to uncover how oxytocinergic modulation within LS circuits orchestrates socially transmitted feeding preferences.

## Results

### Female mice display a more robust STFP performance

Firstly, innate food preference was tested in male and female mice by simultaneously exposing mice to two pairs of scented food: cinnamon- versus cocoa-scented food, and cumin- versus thyme-scented food (Fig 1A). Consumption of each scented food was measured over 1 h. Both male and female mice exhibited a robust innate preference for cocoa over cinnamon (Male: ^*^P = 0.027, paired *t* test; Female: ^**^P = 0.0034, paired *t* test) and for thyme over cumin (Male: ^**^P = 0.0059, Wilcoxon matched-pairs signed rank test; Female: ^**^P = 0.0047, Wilcoxon matched-pairs signed rank test, Fig 1B).

**Fig 1:**
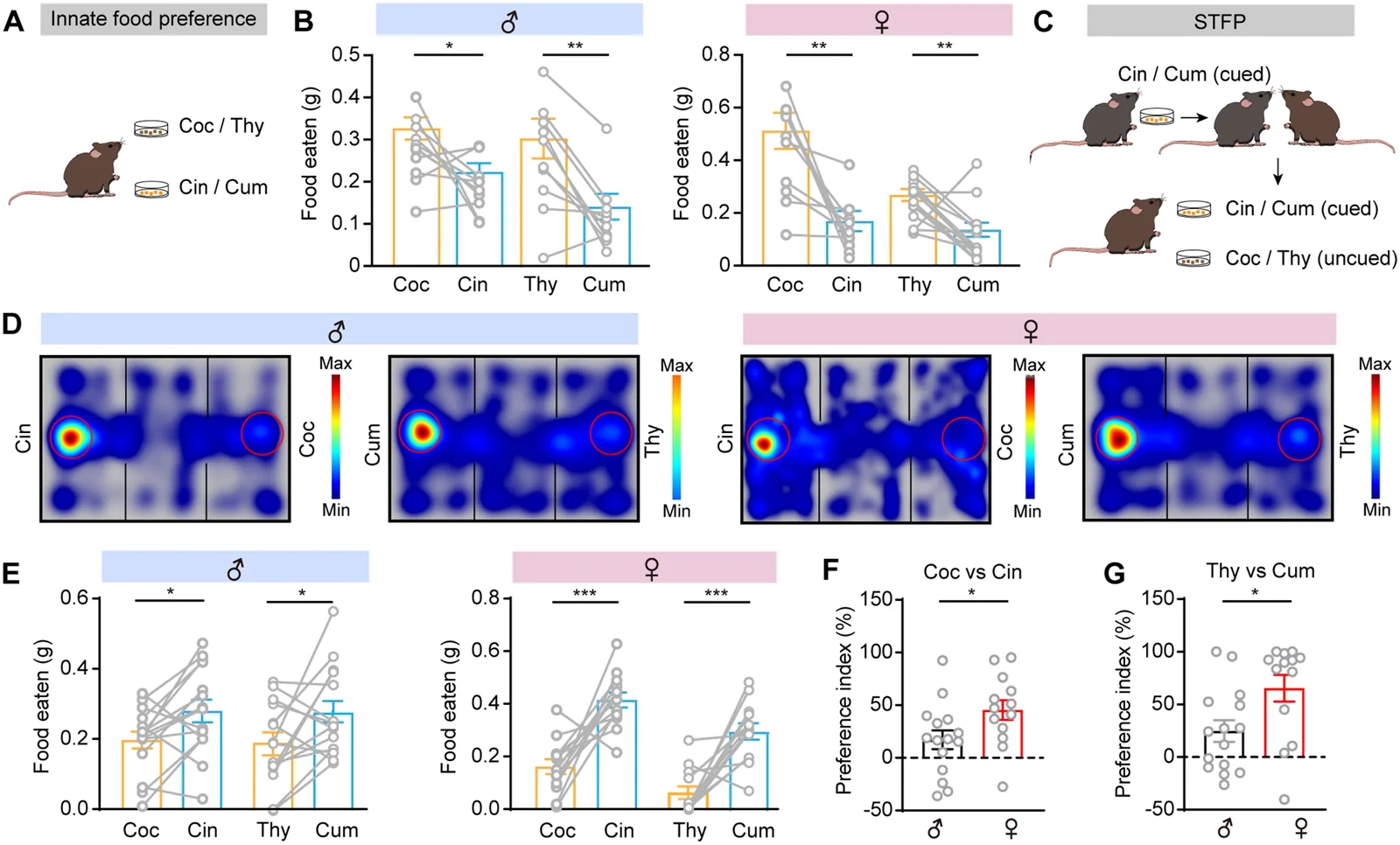
Female mice exhibit a more robust STFP performance. **(A)** Schematic illustration of the innate food preference test. **(B)** Summary plot of food consumption in the innate preference test for male and female mice. Male: Cin vs. Coc: ^*^P = 0.027, paired *t* test, *t*_(10)_ = 2.59, Cohen’s *d* = –0.78, 95% CI [–0.16, –0.012]; Cum vs. Thy: ^**^P = 0.0059, Wilcoxon matched-pairs signed rank test, *W* = –51, *r*_rb_ = –0.93, 95% CI [–0.23, –0.01]; Female: Cin vs. Coc: ^**^P = 0.0034, paired *t* test, *t*_(9)_ = 3.93, Cohen’s *d* = –1.24, 95% CI [–0.46, –0.12]; Cum vs. Thy: ^**^P = 0.0047, Wilcoxon matched-pairs signed rank test, *W* = –125, r_*rb*_ = –0.73, 95% CI [–0.21, –0.01]. *n* = 11 for male mice, *n* = 10 for female mice. **(C)** Schematic illustration of the STFP. **(D)** Representative heat maps showing the locomotor trajectory during food preference phase of the STFP. **(E)** Summary plot of food consumption in the STFP test for male and female mice. Male: Cin vs. Coc: ^*^P = 0.019, paired *t* test, *t*_(14)_ = 2.65, Cohen’s *d* = 0.68, 95% CI [0.016, 0.15]; Cum vs. Thy: ^*^P = 0.046, paired *t* test, *t*_(14)_ = 2.19, Cohen’s *d* = 0.56, 95% CI [0.0017, 0.17]; Female: Cin vs. Coc: ^***^P = 0.0004, paired *t* test, *t*_(12)_ = 4.91, Cohen’s *d* = 1.36, 95% CI [0.14, 0.36]; Cum vs. Thy: ^**^P = 0.0003, paired *t* test, *t*_(12)_ = 4.97, *r*_rb_ = 0.94, 95% CI [0.035, 0.37]. *n* = 15 for male mice, *n* = 13 for female mice. **(F)** Quantification of preference index toward Cin vs. Coc between male and female mice in STFP test. ^*^P = 0.038, unpaired *t* test, *t*_(26)_ = 2.19, Cohen’s *d* = 0.83, 95% CI [–54.99, –1.68]. **(G)** Quantification of preference index toward Cum vs. Thy between male and female mice in STFP test. ^*^P = 0.037, *U* = 52, *r*_rb_ = 0.53, 95% CI [–86.55, –4.44]. *n* = 15 for male mice, *n* = 13 for female mice. Underlying data can be found in S1 Data.

We next challenged both male and female mice with the STFP assay to directly examine sex-dependent effects on social food preference. During the training stage, a food-restricted demonstrator mouse was allowed to consume food pellets scented with a specific cued food (cinnamon or cumin, the innately nonpreferred food), followed by a 15-min social interaction with an observer mouse. During the test stage, the observer mouse was exposed simultaneously to cued and uncued food (cocoa or thyme, the innately preferred food). Food consumption for each option was then measured over a 1-hour period, and a preference index was calculated as (cued food intake – uncued food intake)/(cued food intake + uncued food intake) × 100% (Fig 1C). We found that both the male and female mice exhibited a robust preference for the cued food (Male: Coc versus Cin: ^*^P = 0.019, paired *t* test; Thy versus Cum: ^*^P = 0.046, paired *t* test; Female: Coc versus Cin: ^***^P = 0.0004, paired *t* test; Thy versus Cum: ^***^P = 0.0003, paired *t* test, Fig 1D–1E). Whereas, total food consumption did not differ between the innate and STFP tests in either sex (Male: Coc versus Cin: *n.s.*, *P* = 0.45, Mann Whitney test; Thy versus Cum: *n.s.*, *P* = 0.25, unpaired *t* test, S1A Fig; Female: Coc versus Cin: *n.s.*, *P* = 0.96, unpaired *t* test; Thy versus Cum: *n.s.*, *P* = 0.58, unpaired *t* test, S1B Fig), indicating that social interaction altered preference without affecting total food intake. Although a preference for the cued food was observed in both sexes, we found that female mice exhibited a stronger preference, as indicated by a significantly higher preference index compared to males (Coc versus Cin: ^*^P = 0.038, unpaired *t* test, Fig 1F; Thy versus Cum: ^*^P = 0.037, Mann–Whitney test, Fig 1G). This finding aligns with prior evidence of female-specific behavior sensitivity in STFP [19]. To assess the durability of socially induced food preference, we retested the mice following a 24-hour interval. Although both male and female mice exhibited a robust preference immediately after training (0 hour), this preference was no longer significant at the 24-hour time point (Food consumption of male mice: 0h: ^*^P = 0.043, paired *t* test; 24 hour: *n.s.*, *P* = 0.29, paired *t* test; Preference index: ^*^P = 0.041, paired *t* test, S2A Fig; Food consumption of female mice: 0 hour: ^***^P = 0.0009, paired *t* test; 24 hour: ^**^P = 0.0056, paired *t* test; Preference index: ^****^P < 0.0001, paired *t t*est, S2B Fig).

These results demonstrate that food preference is robustly transmitted through social interaction in both sexes. Notably, the social transmission effect is significantly stronger in females. Given the more robust behavioral profile of female mice in the STFP paradigm, coupled with the need to avoid aggression-related confounds more common in males [20], subsequent experiments were conducted exclusively in female mice.

### LS neural activity distinguishes cued from uncued food in STFP

To identify brain regions that mediate STFP, we performed whole-brain c-Fos immunostaining. Female mice were euthanized at 90 min post-STFP test for quantification of c-Fos-immunoreactive cells. Consistent with the olfactory-dependent nature of STFP, we detected robust c-Fos expression in multiple olfactory processing regions, including the olfactory bulb, anterior olfactory nucleus, and anterior piriform cortex, which was consistent with previous studies [3,6]. Elevated c-Fos expression was also observed in the LS, hippocampus, and lateral hypothalamus (Fig 2A–2B). Given the established roles of LS in social memory and feeding regulation, along with the observed differences in STFP performance between male and female mice, we further compared the c-Fos expression in this region between male and female mice. STFP exposure elicited a pronounced sex-dependent difference in LS c-Fos expression (^****^P < 0.0001, unpaired *t t*est, Fig 2C), with female mice showing significantly higher c-Fos levels, consistent with their stronger behavioral performance to STFP. Together, the LS is differentially recruited during STFP in a sex-dependent manner, suggesting its potential involvement in STFP.

**Fig 2:**
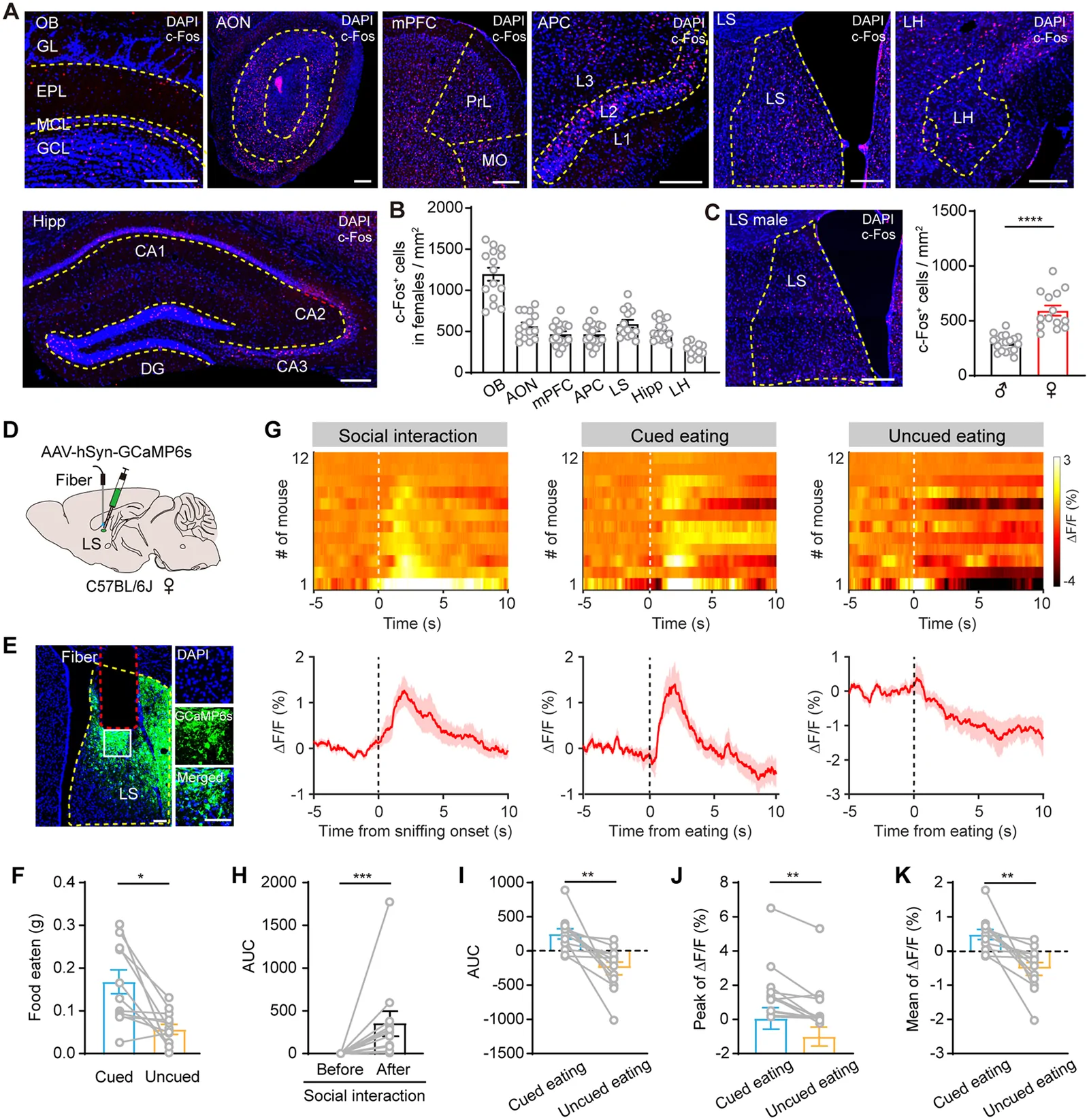
The calcium activity of LS neurons discriminates cued from uncued food during the STFP. **(A)** Representative images of c-Fos immunostaining across key brain regions in female mice following STFP. *n* = 3 mice. Scale bar = 200 μm. **(B)** Average number of c-Fos^+^ cells in different brain regions of female mice. Each dot represents the number of c-Fos^+^ neurons per mm^2^ in a slice. *n* = 6-8 slices from 3 mice. **(C)** Left: a representative image of c-Fos immunostaining in the LS of male mice following STFP. Scale bar = 200 μm. Right: quantitative analysis of c-Fos^+^ cells in the LS between male and female mice. ^****^P < 0.0001, unpaired *t t*est, *t*_(31)_ = 6.63, Cohen’s *d* = 2.33, 95% CI [–385.6, –204.1]. Each do*t* represents the number of c-Fos^+^ neurons per mm^2^, quantified from 11–19 randomly selected fields across 6–8 sections per mouse (*n* = 3 mice per group). **(D)** AAV-hSyn-GCaMP6s was injected into the LS of C57BL/6J mice for calcium signal recording. **(E)** A representative image showing GCaMP6s expression and the fiber placement within the LS. Scale bar = 100 μm. **(F)** Behavioral assessments of mice during calcium activity recordings. Cued vs. uncued: ^**^P = 0.0042 unpaired *t t*est, *t*_(11)_ = 3.60, Cohen’s *d* = –1.099, 95% CI [–0.18, –0.044]. **(G)** Hea*t* maps (top) and averaged traces (bottom) illustrating the calcium responses during the three sessions. **(H)** Quantification of the AUC of the calcium signal during social interaction. ^***^P = 0.001, Wilcoxon matched-pairs signed rank test, *W* = 66, *r*_rb_ = 1.0, 95% CI [84.69, 379.5]. **(I)** Quantification of the AUC of the calcium signal during cued and uncued food eating. ^**^P = 0.0029, paired *t t*est, *t*_(11)_ = 3.81, Cohen’s *d* = –1.099, 95% CI [–794.3, –212.4]. **(J–K)** The Mean and Peak calcium response of LS neurons during cued and uncued food ea*t*ing. Peak ΔF/F, ^**^P = 0.0024, Wilcoxon matched-pairs signed rank test, W = –72, *r*_rb_ = –0.92, 95% CI [–1.59, –0.083]; mean ΔF/F, ^**^P = 0.0029, paired *t t*est, *t*_(11)_ = 3.81, Cohen’s *d* = –1.1, 95% CI [–1.59, –0.43]. *n* = 12 mice. Underlying da*t*a can be found in S1 Data.

To further delineate the dynamic engagement of LS in STFP, we employed fiber photometry to record real-time calcium activity changes in LS neurons of female mice. The AAV-hSyn-GCaMP6s virus was unilaterally injected into the LS of C57BL/6J mice, followed by fiber implantation (Fig 2D). Histological analysis showed that robust expression of GCaMP6s was detected in the LS (Fig 2E). Behavioral results indicated that these manipulations did not affect the normal preference for cued odor (^**^P = 0.0042 unpaired *t* test, Fig 2F). Real-time calcium dynamics of LS neurons during STFP were monitored (Fig 2G). We found that LS neurons exhibited increased calcium activity during social interaction. A significant increase was observed in the area under the curve (AUC) of calcium transients during social interaction (^***^P = 0.001, Wilcoxon matched-pairs signed rank test, Fig 2H). We further compared calcium dynamics across different types of social interaction and found that calcium transients increased significantly during both nose-to-nose contact and anogenital sniffing (S3A*–*S3B Fig). The AUC of calcium signals was significantly elevated during both types of interaction (nose-to-nose contact: ^***^P = 0.0005, Wilcoxon matched-pairs signed rank test, S3C Fig; anogenital sniffing: ^**^P = 0.0034, Wilcoxon matched-pairs signed rank test, S3D Fig), whereas no significant difference between the two interaction types (*n.s.*, *P* = 0.89, Mann Whitney test, S3E Fig). Notably, cued-food eating triggered a robust increase in calcium transients, whereas uncued-food eating led to a sustained decrease in calcium signals (Fig 2G). The AUC during cued-food eating was significantly greater than that during uncued-food eating (^**^P = 0.0029, paired *t* test, Fig 2I). This difference was further confirmed by both peak and mean ΔF/F values (peak ΔF/F: ^**^P = 0.0024, Wilcoxon matched-pairs signed rank test; mean ΔF/F: ^**^P = 0.0029, paired *t* test, Fig 2J*–*2K). To assess potential habituation or satiety effects, we compared the calcium transients by plotting all trials of cued and uncued eating and comparing calcium responses between the first trial and last trial across all mice (S3F*–*S3G Fig). No significant difference were observed in either peak or mean ΔF/F between the first and last trial for both cued food (Peak ΔF/F: *n.s.*, *P* = 0.47, Wilcoxon matched-pairs signed rank test; Mean ΔF/F: *n.s.*, *P* = 0.57, Wilcoxon matched-pairs signed rank test, S3H-S3J Fig) and uncued food (Peak ΔF/F: *n.s.*, *P* = 0.85, Wilcoxon matched-pairs signed rank test; Mean ΔF/F: *n.s.*, *P* = 0.85, Wilcoxon matched-pairs signed rank test, S3K*–*S3M Fig), indicating that the calcium signals remained stable throughout the session and were not confounded by progressive changes in motivation or satiety.

Given the distinct behavioral performance between male and female mice in the STFP, we next examined whether LS calcium dynamics also exhibit sex differences. To this end, we performed the same recordings in male mice under identical experimental conditions (S4A*–*S4B Fig). Behaviorally, the mice showed normal preference for cued odor (^*^P = 0.011, paired *t* test, S4C Fig). Notably, the calcium dynamics in male LS neurons closely paralleled those observed in females, exhibiting similarly significant increases during social interaction (AUC of social interaction: ^**^P = 0.0097, paired *t* test, S4D–S4E Fig), while showing differential responses during cued versus uncued food consumption (AUC of food eating: ^*^P = 0.027, paired *t* test; Peak ΔF/F: ^*^P = 0.029, paired *t* test; Mean ΔF/F: ^*^P = 0.027, paired *t* test, S3D and S4F–S4H Figs). These results indicate that LS neurons activity undergoes experience-dependent plasticity during STFP, with opposing response patterns to cued and uncued food. Collectively, these findings indicate that LS neurons can discriminate between socially cued and uncued food, supporting the crucial involvement of the LS in STFP.

To investigate the LS neuronal activity in response to different stages during STFP behavior at the single-cell level, we recorded the spike signals from the LS in mice performing the STFP test. Microelectrodes were implanted into the LS (S5A Fig) to recorded extracellular single-unit activity of LS neurons during STFP behavior. The behavioral results confirmed that mice implanted with microelectrodes exhibited a preference for cued food in the STFP test (^*^P = 0.033, paired *t* test, S5B Fig). As shown in S5C Fig, firing activity of LS neurons was recorded and single units could be clearly sorted from the raw spikes. To quantify the spike responses during STFP, we analyzed the normalized mean firing rate (MFR). The results showed a slight increase in firing rates during the social interaction stage (S5D Fig). Notably, the firing rates increased during cued-food eating (S5E Fig), but remained unchanged during uncued-food eating (S5F Fig). When comparing normalized MFR between cued- and uncued-food eating, we found a significantly higher firing rate during cued-food eating across all recorded units (Cumulative probability: ^**^P = 0.0054, Kolmogorov-Smirnov test; normalized MFR: ^**^P = 0.0017, Wilcoxon matched-pairs signed rank test, S5G*–*S5I Fig), which is consistent with the calcium recording results.

It’s reported that local field potential (LFP) spectral bands in the LS reflect the neuronal network activity [8]. We therefore analyzed LFP power oscillations during the STFP task. The results indicated no significant differences in theta, beta, low-gamma or high-gamma power between cued and uncued-food eating (theta oscillations: *n.s.*, *P* = 0.36, unpaired *t* test, S6A*–*S6C Fig; beta oscillations: *n.s.*, *P* = 0.37, unpaired *t* test, S6D*–*S6F Fig; low gamma oscillations: *n.s.*, *P* = 0.22, unpaired *t* test, S6G*–*S6I Fig; high gamma oscillations: *n.s.*, *P* = 0.80, Mann–Whitney test, S6J*–*S6L Fig).

Taken together, these findings indicate that LS calcium dynamics and firing activity differentiate cued and uncued food, serving as neural correlates of the socially assigned value of food.

### LS specifically modulate social learning of STFP

To determine whether LS neuronal activity is required for STFP, we first employed chemogenetic approaches to inactivate LS neurons and evaluate their functional necessity in STFP. Expression of hM4Di was induced in bilateral LS of C57BL/6J mice through AAV-hSyn-hM4Di-mCherry virus injection (Fig 3A). The effect of clozapine N-oxide (CNO) on the activity of hM4Di-expressing neurons was evaluated *in vitro*. CNO (10 μM) perfusion significantly reduced the firing rate of hM4Di-positive neurons in the LS (^***^P = 0.0006, paired *t* test, Fig 3B). The STFP test was conducted 45 min post saline or CNO administration (Fig 3C). As shown in Fig 3D*–*3E, hM4Di + saline-treated mice displayed a strong preference for cued food (Cin versus Coc: ^**^P = 0.0034, Wilcoxon matched-pairs signed rank test, Fig 3D Left; Cum versus Thy: ^**^P = 0.0077, Wilcoxon matched-pairs signed rank test, Fig 3E Left). In contrast, hM4Di + CNO-treated mice reversed this preference, favoring uncued food instead (Cin versus Coc: ^**^P = 0.0018, paired *t* test. Fig 3D Left; Cum versus Thy: ^*^P = 0.033, Wilcoxon matched-pairs signed rank test, Fig 3E Left). The total food consumption was not significantly different between the two groups (Cin versus Coc: *n.s.*, *P* = 0.86, Mann–Whitney test; Cum versus Thy: *n.s.*, *P* = 0.34, unpaired *t* test. S7A Fig), excluding the possibility that altered appetite or general motivation contributed to the observed shift in food preference. We then analyzed the preference index for cued food and observed a significant reduction in hM4Di+CNO-treated mice compared to control groups (Cin versus Coc: ^****^P < 0.0001, Mann–Whitney test; Cum versus Thy: ^**^P = 0.0023, Mann–Whitney test, Fig 3D Right and 3E Right), indicating that LS is necessary for STFP expression.

**Fig 3:**
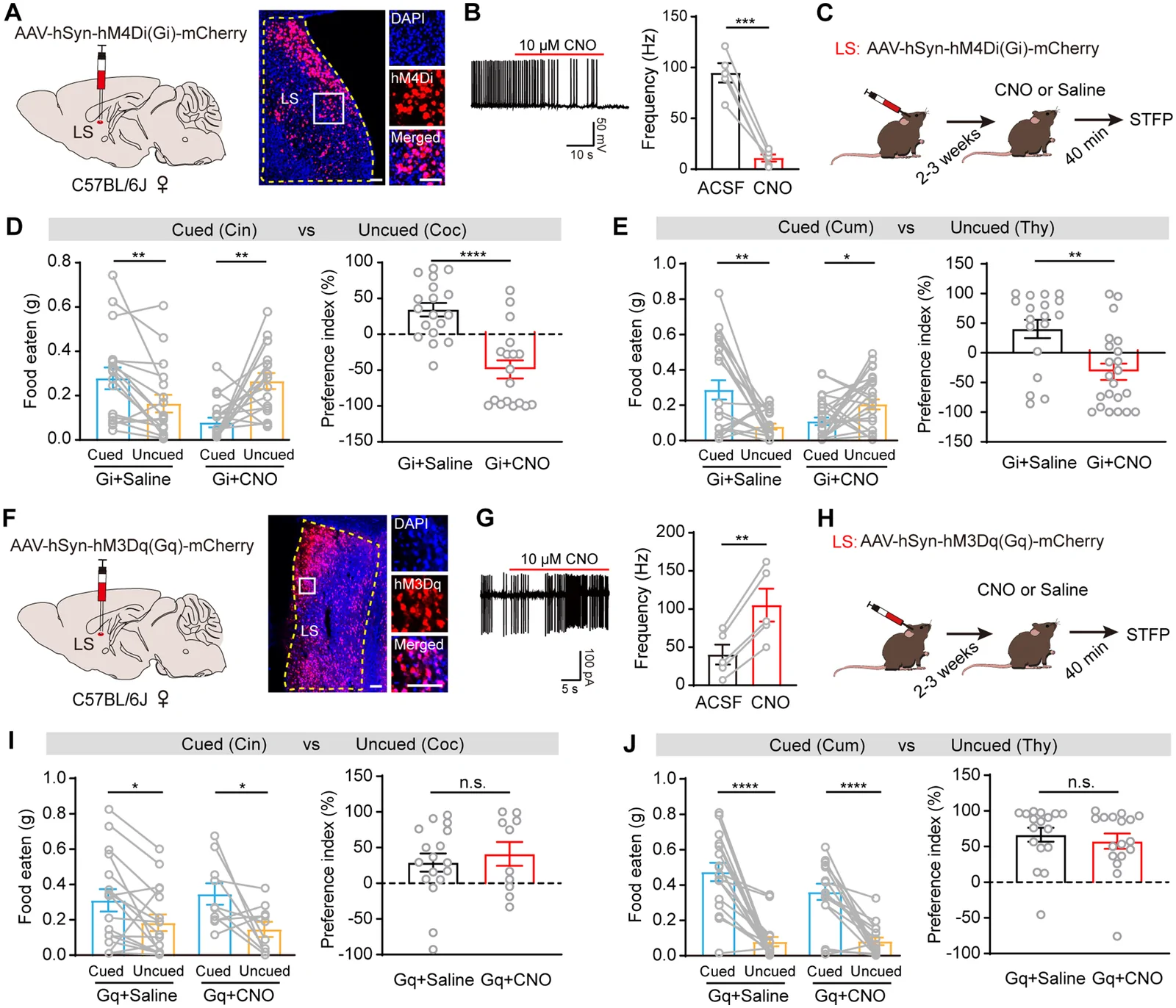
LS activity is necessary for the formation of STFP. **(A)** Left: schematic diagram of AAV-hSyn-hM4Di(Gi)-mCherry injection into the LS of C57BL/6 female mice. Right: representative images showing hM4Di-mCherry expression in the LS. Scale bar = 100 μm. **(B)** Left: Effect of CNO perfusion on action potential firing in an hM4Di-expressing LS neuron. Right: Quantitative analysis of CNO effect on the spontaneous firing activity of hM4Di-expressing neurons. ^***^P = 0.0006, paired *t t*est, *t*_(4)_ = 9.78, Cohen’s *d* = –4.37, 95% CI [–106.6, –59.42], *n* = 5 cells form 3 mice. **(C)** Schema*t*ic illustration of the experimental timeline. **(D)** Chemogenetic inactivation of LS neurons impaired the preference for the cued cinnamon during the STFP. Left: food consumption, Gi+saline: ^**^P = 0.0034, Wilcoxon matched-pairs signed rank test, *W* = –129, *r*_rb_ = –0.75, 95% CI [–0.21, –0.014]; Gi + CNO: ^**^P = 0.0018, paired *t t*est, *t*_(17)_ = 3.70, Cohen’s *d* = 0.87, 95% CI [0.080, 0.29]. Righ*t*: preference index, Gi+saline vs. Gi + CNO: ^****^P < 0.0001, Mann-Whitney test, *U* = 36, *r*_rb_ = 0.78, 95% CI [–119, –56.63]. **(E)** Chemogenetic inactivation of LS neurons impaired the preference for the cued cumin during the STFP. Left: food consumption, Gi+saline: ^**^P = 0.0077, Wilcoxon matched-pairs signed rank test, *W* = –119, *r*_rb_ = –0.70, 95% CI [–0.52, –0.01]; Gi + CNO: ^*^P = 0.033, Wilcoxon matched-pairs signed rank test, *W* = 131, *r*_rb_ = –0.52, 95% CI [–0.05, 0.28]. Right: preference index, Gi+saline vs. Gi + CNO: ^**^P = 0.0023, Mann-Whitney test, *U* = 88, *r*_rb_ = 0.56, 95% CI [–132.3, –27.32]. *n* = 18 mice for Gi+saline group, *n* = 22 mice for Gi + CNO group. **(F)** Left: schematic diagram of AAV-hSyn-hM3Dq(Gq)-mCherry injection into the LS of C57BL/6 female mice. Right: representative images showing hM3Dq-mCherry expression in the LS. Scale bar = 100 μm. **(G)** Left: Representative action potential trace recorded from a hM3Dq-expressing LS neuron following CNO application. Right: Quantitative analysis of CNO effect on the spontaneous firing activity of hM3Dq‑expressing neurons. ^**^P = 0.0017, paired *t* tes*t*, *t*_(4)_ = 7.55, Cohen’s *d* = 3.37, 95% CI [40.7, 80.1], *n* = 5 cells form 3 mice. **(H)** Schematic illus*t*ration of the experimental timeline. **(I)** Chemogenetic activation of LS neurons did not affect the preference for the cued cinnamon during the STFP. Left: food consumption, Gi+saline: ^*^P = 0.013, Wilcoxon matched-pairs signed rank test, *W* = –103, *r*_rb_ = –0.67, 95% CI [–0.25, –0.011]; Gq + CNO: ^*^P = 0.045, paired *t* tes*t*, *t*_(9)_ = 2.33, Cohen’s *d* = –0.74, 95% CI [–0.39, –0.0055]. Right: preference index, Gq+saline vs. Gq + CNO: *n.s.*, *P* = 0.57, unpaired *t* tes*t*, *t*_(25)_ = 0.58, Cohen’s *d* = 0.23, 95% CI [–30.97, 55.24]. **(J)** Chemogenetic ac*t*ivation of LS neurons did not affect the preference for the cued cumin during the STFP. Left: food consumption, Gq+saline: ^****^P < 0.0001, Wilcoxon matched-pairs signed rank test, *W* = –151, *r*_rb_ = –0.99, 95% CI [–0.60, –0.19]; Gq + CNO: ^****^P < 0.0001, Wilcoxon matched-pairs signed rank test, *W* = –147, *r*_rb_ = –0.96, 95% CI [–0.49, –0.14]. Right: preference index, Gq+saline vs. Gq + CNO: *n.s.*, *P* = 0.43, Mann-Whitney test, *U* = 121, *r*_rb_ = 0.16, 95% CI [–38.37, 10.71], Cohen’s d = –1.1, 95% CI [–1.59, –0.43]. *n* = 17 mice for Gq+saline group, *n* = 10 mice for Gq + CNO group. Underlying data can be found in S1 Data.

To further determine whether increased LS neuronal activity affects STFP, we injected AAV-hSyn-hM3Dq-mCherry in the bilateral LS of C57BL/6J mice (Fig 3F). Application of CNO (10 μM) markedly increased the firing rate of hM3Dq-positive neurons in the LS (^**^P = 0.0017, paired *t* test, Fig 3G). We then performed the STFP test to assess the behavioral consequence of this enhanced activity (Fig 3H). Both hM3Dq+saline- and hM3Dq + CNO-treated mice exhibited a significant preference for the cued food (Cin versus Coc: Gi+saline: ^*^P = 0.013, Wilcoxon matched-pairs signed rank test; Gq + CNO: ^*^P = 0.045, paired *t* test, Fig 3I Left; Cum versus Thy: Gq+saline: ^****^P < 0.0001, Wilcoxon matched-pairs signed rank test; Gq + CNO: ^****^P < 0.0001, Wilcoxon matched-pairs signed rank test, Fig 3J Left), and the preference indices did not differ between the two groups (Cin versus Coc: *n.s.*, *P* = 0.57, unpaired *t* tes*t*, Fig 3I Right; Cum versus Thy: *n.s.*, *P* = 0.43, Mann–Whitney test, Fig 3J Right). Similarly, total food consumption was not significantly different between groups (Cin versus Coc: *n.s.*, *P* = 0.96, unpaired *t* tes*t*; Cum versus Thy: *n.s.*, *P* = 0.18, Mann–Whitney test, S7B Fig). These results indicate that chemogenetic activation of LS neurons does not alter the expression of STFP.

To further examine whether LS neurons regulate STFP by modulating social memory, we performed optogenetic inhibition to selectively and acutely suppress LS neuron activity during the social learning phase of the STFP task. GtACR1 expression was induced in bilateral LS of C57BL/6J mice via AAV-hSyn-GtACR1-EGFP injection (S8A Fig). Whole-cell recordings confirmed light-induced inhibition of the GtACR1-positive neuron (S8B Fig). Photostimulation (20 Hz) was exclusively applied to observer mice during the social learning phase to inhibit LS neurons. Optogenetic inhibition of the LS during social learning significantly decreased the preference index (food consumption of Cin versus Coc, EGFP: ^**^P = 0.0090, paired *t* test; GtACR1: *n.s.*, *P* = 0.22, Wilcoxon matched-pairs signed rank test; preference index of Cin versus Coc: ^*^P = 0.044, unpaired *t* test, S8C Fig; food consumption of Cum versus Thy, EGFP: ^*^P = 0.023, Wilcoxon matched-pairs signed rank test; GtACR1: *n.s.*, *P* = 0.64, Wilcoxon matched-pairs signed rank test; preference index of Cum versus Thy: *n.s.*, *P* = 0.19, Mann Whitney test, S8D Fig). This result indicates that inhibition of LS neurons impairs STFP, likely by disrupting social learning processes. To examine whether LS neuron activity is generally required for social learning, we next performed a three‑chamber social interaction test under optogenetic inhibition of LS neurons. In the sociablity test (S8E Fig), both control (EGFP) and optogenetic inhibition (GtACR1) groups showed a significant preference for a stimulus mouse over an empty enclosure (EGFP: ^**^P = 0.0078, Wilcoxon matched-pairs signed rank test; GtACR1: ^**^P = 0.0044, paired *t* test. S8F Fig). The discrimination index did not differ between groups (*n.s.*, *P* = 0.96, Mann–Whitney test. S8G Fig). In the social novelty test (S8H Fig), both groups exhibited a significant preference for a novel mouse over a familiar one (EGFP: ^*^P = 0.029, paired *t* test; GtACR1: ^**^P = 0.0078, Wilcoxon matched-pairs signed rank test. S8I Fig), with no between‑group difference in the discrimination index (*n.s.*, *P* = 0.69, paired *t* test. S8J Fig). These results indicate that LS neurons regulate STFP through specific modulation of social learning.

### dLS GABAergic neurons are essential for regulating STFP

LS is principally composed of GABAergic neurons, with dLS and vLS displaying distinct neuronal composition and functional profiles [18,21,22]. To further dissect the contribution of neuronal activity within these subregions to STFP, we firstly injected AAV-Vgat-hM4Di-mCherry into dLS (Fig 4A). The STFP test was conducted 45-minutes post CNO or saline administration (Fig 4B). As shown in Fig 4C, hM4Di+saline-treated mice displayed a robust preference for the cued food, indicating intact STFP (Cin versus Coc: ^*^P = 0.017, paired *t* test; Cum versus Thy: ^*^P = 0.027, paired *t* test. Fig 4C*–*4D Left). In contrast, hM4Di+CNO-treated mice exhibited a preference for the uncued food, which suggests an impairment of STFP (Cin versus Coc: Gi + CNO: ^*^P = 0.030, paired *t* test; Cum versus Thy: Gi + CNO: ^*^P = 0.016, Wilcoxon matched-pairs signed rank test, Fig 4C*–*4D left). In addition, hM4Di+CNO-treated mice showed a significant reduction in the preference index for the cued food compared to the control mice (Cin versus Coc: ^***^P = 0.0008, unpaired *t* test; Cum versus Thy: ^***^P = 0.0009, unpaired *t* test, Fig 4C*–*4D right). We next asked whether GABAergic neurons in vLS similarly contribute to STFP. To this end, AAV-Vgat-hM4Di-mCherry was injected into the vLS of mice (Fig 4E*–*4F). In contrast to the effects observed in the dLS, both hM4Di+saline-treated mice and hM4Di+CNO-treated mice showed a preference for the cued food (food consumption of Cin versus Coc, Gi+saline: ^*^P = 0.021, paired *t* test; Gi + CNO: ^*^P = 0.023, Wilcoxon matched-pairs signed rank test. Fig 4G left; food consumption of Cum versus Thy, Gi+saline: ^*^P = 0.039, Wilcoxon matched-pairs signed rank test; Gi + CNO: *n.s.*, *P* = 0.062, paired *t* test. Fig 4H left). Furthermore, the preference indices did not differ significantly between the two groups (preference index of Cin versus Coc: *n.s.*, *P* = 0.10, Mann–Whitney test, Fig 4G right; preference index of Cum versus Thy: *n.s.*, *P* = 0.73, unpaired *t* test, Fig 4H right). Together, these results demonstrate that the dLS, but not the vLS, is necessary for STFP.

**Fig 4:**
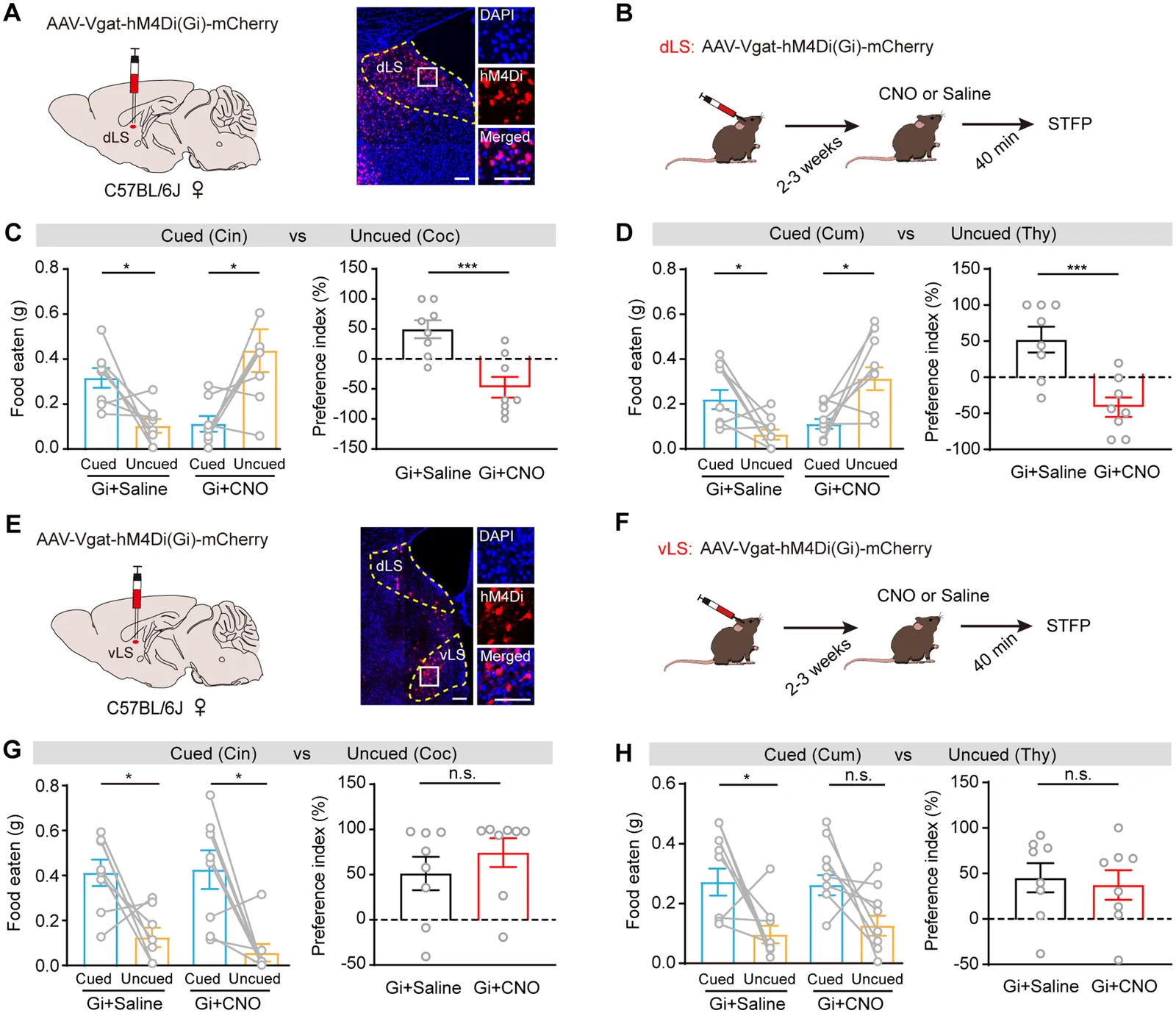
The dLS GABAergic neurons are required for the STFP. **(A)** Left: AAV-Vgat-hM4Di(Gi)-mCherry injection into the dLS of C57BL/6 female mice. Right: representative images showing hM4Di-mCherry expression in the dLS. Scale bar = 100 μm. **(B)** Schematic diagram of experimental procedures. **(C)** Chemogenetic inactivation of dLS neurons impaired the preference for the cued cinnamon during the STFP. Left: food consumption, Gi+saline: ^*^P = 0.017, paired *t* test, *t*_(7)_ = 3.13, Cohen’s *d* = –1.11, 95% CI [–0.38, –0.052]; Gi + CNO: ^*^P = 0.030, paired *t* test, *t*_(7)_ = 2.72, Cohen’s *d* = 0.96, 95% CI [0.043, 0.61]. Righ*t*: preference index, Gi+saline vs. Gi + CNO: ^***^P = 0.0008, unpaired *t* tes*t*, *t*_(14)_ = 4.24, Cohen’s *d* = 2.12, 95% CI [–145.7,–47.75]. **(D)** Chemogene*t*ic inactivation of dLS neurons impaired *t*he preference for the cued cumin during the STFP. Food consumption: Gi+saline: ^*^P = 0.027, paired *t* test, *t*_(7)_ = 2.78, Cohen’s *d* = –0.98, 95% CI [–0.33, –0.027]; Gi + CNO: ^*^P = 0.016, paired *t* test, *t*_(7)_ = 3.15, Cohen’s *d* = 1.11, 95% CI [0.058, 0.41]. Righ*t*: preference index, Gi+saline vs. Gi + CNO: ^***^P = 0.0009, unpaired *t* tes*t*, *t*_(14)_ = 4.17, Cohen’s *d* = 2.086, 95% CI [–141.8, –45.48]. *n* = 8 mice for each group. **(E)** Lef*t*: AAV-Vgat-hM4Di(Gi)-mCherry injection in*t*o the vLS of C57BL/6 female mice. Right: representative images showing hM4Di-mCherry expression in the vLS. Scale bar = 100 μm. **(F)** Schematic diagram of experimental procedures. **(G)** Chemogenetic inactivation of vLS neurons did not affect the preference for the cued cinnamon during the STFP. Left: food consumption, Gi+saline: ^*^P = 0.021, paired *t* test, *t*_(7)_ = 2.95, Cohen’s *d* = –1.044, 95% CI [–0.52, –0.057]; Gi + CNO: ^*^P = 0.023, Wilcoxon ma*t*ched-pairs signed rank test, *W* = –32, *r*_rb_ = –0.89, 95% CI [–0.75, 0.10]. Righ*t*: preference index, Gi+saline vs. Gi + CNO: *n.s.*, *P* = 0.10, Mann Whitney test, *U* = 16, *r*_rb_ = 0.50, 95% CI [–3.98, 64.54]. **(H)** Chemogenetic inactivation of vLS neurons did not affect the preference for the cued cumin during the STFP. Food consumption: Gi+saline: ^*^P = 0.039, Wilcoxon matched-pairs signed rank test, *W* = –30, *r*_rb_ = –0.83, 95% CI [–0.45, 0.18]; Gi + CNO: *n.s.*, *P* = 0.062, paired *t* test, *t*_(7)_ = 2.22, Cohen’s *d* = –0.78, 95% CI [–0.33, 0.010]. Righ*t*: preference index, Gi+saline vs. Gi + CNO: *n.s.*, *P* = 0.73, unpaired *t* tes*t*, *t*_(14)_ = 0.35, Cohen’s *d* = 0.17, 95% CI [–56.77, 41.02]. *n* = 8 mice for each group. Underlying da*t*a can be found in S1 Data.

To further delineate the engagement of vLS in STFP, we used fiber photometry to record vLS neuronal calcium activity during STFP by administering AAV-hSyn-GCaMP6s into the vLS of C57BL/6J mice (S9A–S9B Fig). Behaviorally, mice showed a significant preference for the cued food (^**^P = 0.0098, Wilcoxon matched-pairs signed rank test, S9C Fig). Calcium signals in the vLS remained stable throughout the STFP task (S9D Fig). It exhibited no significant change during social interaction (AUC: *P* = 0.16, paired *t* test, S9E Fig) and no significant difference between cued and uncued eating periods (AUC: *P* = 0.38, paired *t* test; Peak ΔF/F: *P* = 0.11, Wilcoxon matched-pairs signed rank test; Mean ΔF/F: *P* = 0.63, Wilcoxon matched-pairs signed rank test, S9F–S9H Fig). This stability in the vLS activity aligns with the behavioral result that the vLS is not required for STFP.

Thus, these results demonstrate a subregion-specific requirement for LS GABAergic neurons in STFP and indicate that the dLS, but not the vLS, is necessary for STFP regulation.

### Specific oxytocin receptor signaling in the dLS promotes STFP

Oxytocin and vasopressin within the LS critically regulate diverse social behaviors [23,24], and rodents exhibit highly specific expression patterns of the oxytocin receptor (OXTR) and AVP V1a receptor (V1aR) across LS subregions [24,25]. To determine whether OXTR or V1aR signaling differentially modulates STFP, we implanted a cannula into the lateral ventricle for intracerebroventricular (i.c.v.) antagonist delivery (Fig 5A–5B). Control mice infused with artificial cerebrospinal fluid (ACSF) maintained a preference for the cued Cinnamon (^**^P = 0.0021, Wilcoxon matched-pairs signed rank test, Fig 5C left). In contrast, i.c.v. administration of the OXTR antagonist atosiban (0.02 mM) abolished this preference (*n.s.*, *P* = 0.064, paired *t* test, Fig 5C left). The preference index of the atosiban group was significantly lower than that in the control group (^**^P = 0.0065, Mann–Whitney test. Fig 5C right). This impairing effect of atosiban was further confirmed in the Cum versus Thy food test (Food consumption: ACSF: ^**^P = 0.0031, Wilcoxon matched-pairs signed rank test; Atosiban: *n.s.*, *P* = 0.18, Wilcoxon matched-pairs signed rank test; Preference index: ^**^P = 0.0029, Mann–Whitney test. Fig 5D), indicating that oxytocin signaling is critical for the regulation of STFP. We then administrated a V1aR antagonist (0.03 mM) to examined whether V1aR signaling is required for STFP. The results showed that both the ACSF group and the V1aR a group showed a preference for the cued cinnamon (ACSF: ^*^P = 0.016, Wilcoxon matched-pairs signed rank test; V1aR a: ^*^P = 0.025, paired *t* test, S10A Fig left) and cued cumin (ACSF: ^**^P = 0.036, paired *t* test; V1aR a: ^*^P = 0.013, paired *t* test, S10B Fig left). Preference indices did not differ significantly between the two groups (preference index of Cin versus Coc: *n.s.*, *P* = 0.22, Mann–Whitney test, S10A Fig right; preference index of Cum versus Thy: *n.s.*, *P* = 0.33, Mann–Whitney test, S10B Fig right).

**Fig 5:**
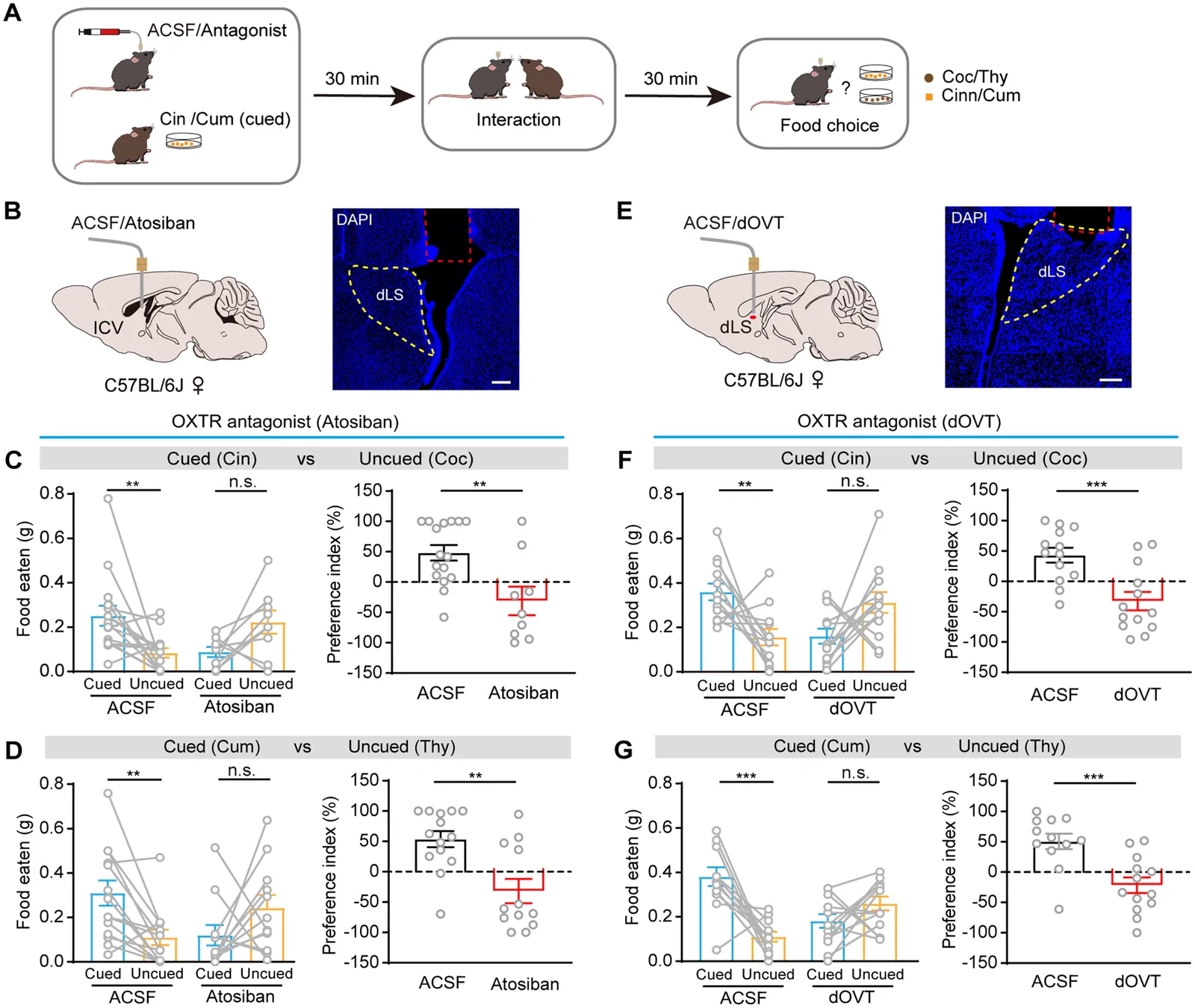
Administration of OXTR antagonist impairs STFP. **(A)** Schematic diagram of experimental procedures. **(B)** Left: schematic diagram of ACSF/Antagonist injection into the lateral ventricle of C57BL/6 mice. Right: representative images showing the cannula placement within the lateral ventricle. Scale bar = 200 μm. **(C)** Administration of OXTR antagonist atosiban abolished the preference for the cued cinnamon during the STFP. Food consumption: ACSF: ^**^P = 0.0021, Wilcoxon matched-pairs signed rank test, *W* = –112, *r*_rb_ = –0.82, 95% CI [–0.30, –0.016]; Atosiban: *n.s.*, *P* = 0.064, paired *t* test, *t*_(8)_ = 2.15, Cohen’s *d* = 0.72, 95% CI [–0.010, 0.28]. Preference index: ^**^P = 0.0065, Mann-Whi*t*ney test, *U* = 25.5, *r*_rb_ = 0.65, 95% CI [–139.3, –29.31]. **(D)** Adminis*t*ration of OXTR antagonist atosiban abolished the preference for the cued cumin during the STFP. Food consumption: ACSF: ^**^P = 0.0031, Wilcoxon matched-pairs signed rank test, *W* = –89, *r*_rb_ = –0.85, 95% CI [–0.33, –0.058]; Atosiban: *n.s.*, *P* = 0.18, Wilcoxon matched-pairs signed rank test, *W* = 36, *r*_rb_ = 0.46, 95% CI [–0.072, 0.34]. Preference index: ^**^P = 0.0029, Mann-Whitney test, *U* = 28, *r*_rb_ = 0.67, 95% CI [–153.3, –30.48]. *n* = 8 mice for each group. n = 16 mice for the ACSF group, *n* = 9 mice for the Atosiban group. **(E)** Left: schematic diagram of ACSF/dOVT injection locally into the dLS of C57BL/6 mice. Right: representative images showing the cannula placement within dLS. Scale bar = 200 μm. **(F)** Administration of dOVT abolished the preference for the cued cinnamon during the STFP. Food consumption: ACSF: ^**^P = 0.0089, paired *t* test, *t*_(12)_ = 3.12, Cohen’s *d* = –0.85, 95% CI [–0.35, –0.062]; dOVT: *n.s.*, *P* = 0.057, paired *t* test, *t*_(12)_ = 2.11, Cohen’s *d* = 0.59, 95% CI [–0.0050, 0.30]. Preference index: *n.s.*, ^***^P = 0.0007, unpaired *t* test, *t*_(24)_ = 3.91, Cohen’s *d* = 1.53, 95% CI [–115.7, –35.75]. **(G)** Adminis*t*ration of dOVT abolished the preference for *t*he cued cumin during the STFP. Food consumption: ACSF: ^***^P = 0.0009, paired *t* test, *t*_(11)_ = 4.49, Cohen’s *d* = –1.29, 95% CI [–0.39, –0.13]; dOVT: *n.s.*, *P* = 0.12, paired *t* test, *t*_(12)_ = 1.67, Cohen’s *d* = 0.46, 95% CI [–0.024, 0.18]. Preference index: ^***^P = 0.0006, unpaired *t* test, *t*_(23)_ = 3.98, Cohen’s *d* = 1.59, 95% CI [–110.1, –34.83]. *n* = 13 mice for each group. Underlying da*t*a can be found in S1 Data.

Given that atosiban can act as a Gi-biased agonist rather than a neutral OXTR antagonist [26,27], we further confirmed that the observed behavioral effect was indeed due to OXTR blockade rather than Gi-biased activity. To this end, we locally administrated a more potent and selective neutral OXTR antagonist, d(CH_2_)^1^_5_, Tyr(Me)^2^, Orn^8-^oxytocin (dOVT, 0.5 mM) [27], into the dLS (Fig 5E). ACSF‑treated controls maintained a robust preference for cued food (Cin versus Coc: ^**^P = 0.0089, paired *t* test, Fig 5F Left; Cum versus Thy: ^***^P = 0.0009, paired *t* test, Fig 5G Left), whereas dOVT administration significantly reduced this preference (Cin versus Coc: *n.s.*, *P* = 0.057, paired *t* test, Fig 5F Left; Cum versus Thy: *n.s.*, *P* = 0.12, paired *t* test, Fig 5G Left), resulting in significantly lower preference indices compared to controls (Cin versus Coc: ^***^P = 0.0007, unpaired *t* test, Fig 5F Right; Cum versus Thy: ^***^P = 0.0006, unpaired *t* test, Fig 5G Right). These results demonstrate that OXTR signaling, but not V1aR signaling, is necessary for STFP, highlighting neuropeptide-specific regulation of STFP. The above results suggest that oxytocin signaling in the dLS may participate in STFP regulation. Previous studies have reported sex-dependent difference in OXTR expression in mice [28,29]. We therefore performed immunofluorescence analysis in the dLS to examine OXTR expression in both sexes. As shown in S11A–S11C Fig, female mice displayed significantly higher mean fluorescence intensity of OXTR and total intensity of OXTR^+^ cells in the dLS compared to males (OXTR mean intensity: ^*^P = 0.012, unpaired *t* test, S11B Fig; total OXTR intensity of OXTR^+^ cells: ^****^P < 0.0001, Mann–Whitney test, S11C Fig). To overcome the limitations of antibody‑based detection, we performed RNAscope in situ hybridization against *Oxtr* mRNA in dLS slices (Fig 6A). This approach confirmed that female mice exhibit significantly higher *Oxtr* transcript density and a greater number of *Oxtr*⁺ cells in the dLS compared to males (*Oxtr* density: ^***^P = 0.0002, unpaired *t t*est; number of *Oxtr*⁺ cells: ^****^P < 0.0001, unpaired *t* tes*t*. Fig 6A–6C). Given the lower OXTR expression in males, we next asked whether exogenous oxytocin could rescue their weaker STFP performance (Fig 6D*–*6E). Local dLS administration of TGOT (0.05 mM) significantly enhanced cued food preference in male mice compared to ACSF controls (Cin versus Coc: ACSF: ^*^P = 0.049, paired *t* tes*t*; TGOT: ^****^P < 0.0001, paired *t* test, Fig 6F Lef*t*; Cum versus Thy: ACSF: ^*^P = 0.026, paired *t* test; TGOT: ^****^P < 0.0001, paired *t* test, Fig 6G Left). Moreover, the TGOT group exhibited significantly higher preference indices compared to the ACSF controls (Cin versus Coc: *n.s.*, *P* = 0.064, unpaired *t* test, Fig 6F Right; Cum versus Thy: ^*^P = 0.041, unpaired *t* test. Fig 6G right). Together, these results indicate that OXTR signaling is essential for STFP and that enhancing OXTR signaling can promote STFP formation in male mice.

**Fig 6:**
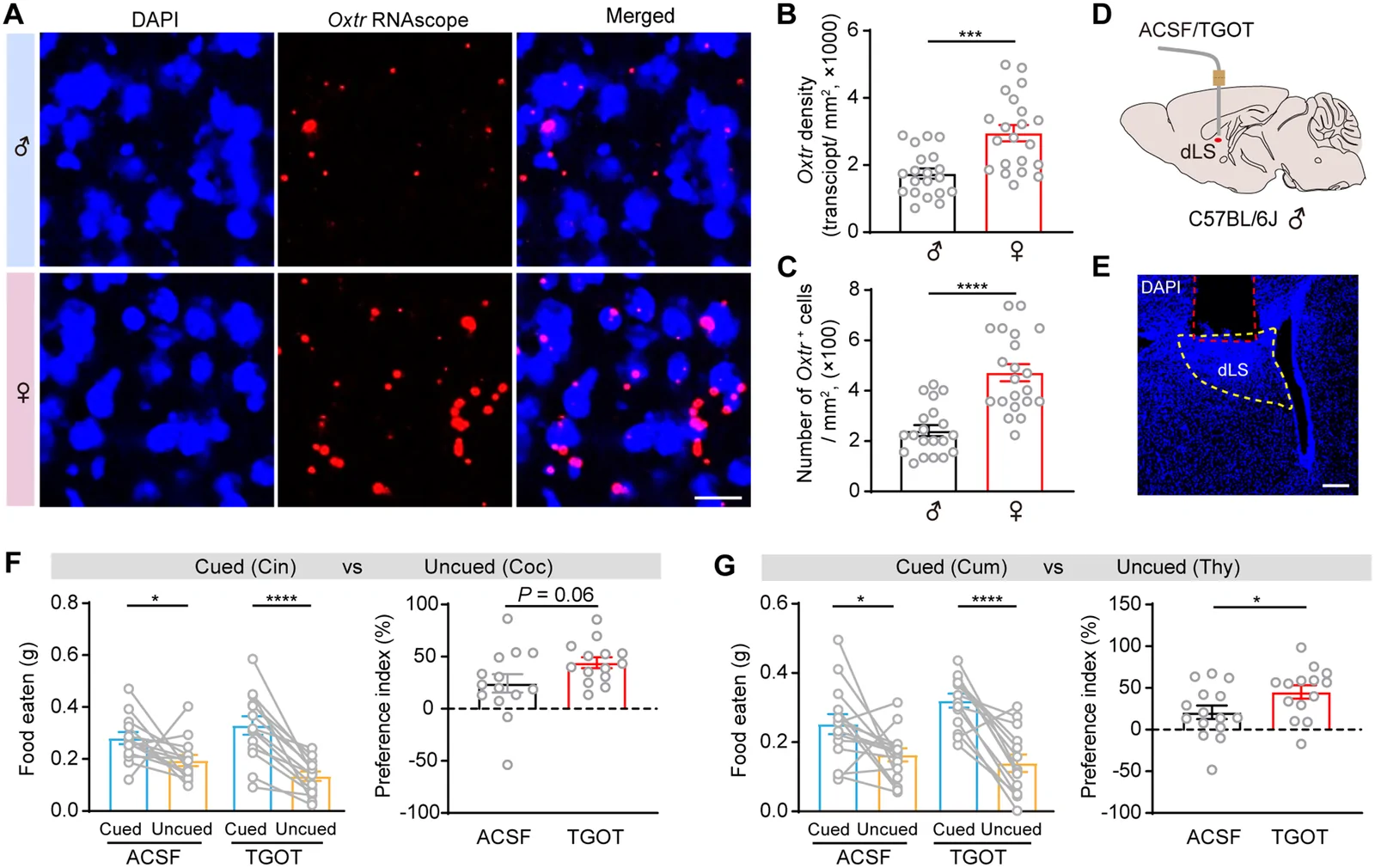
TGOT administration to dLS enhances stability of STFP behavior in male mice. **(A)** Representative images showing RNAscope analysis of *Oxtr* expression in the dLS of male and female mice. Scale bar = 20 μm. **(B)** Quantitative analysis of *Oxtr* density in the dLS between male and female mice. ^***^P = 0.0002, unpaired *t* test, *t*_(39)_ = 4.18, Cohen’s *d* = 1.31, 95% CI [621.8, 1,786]. **(C)** Quantitative analysis of number of *Oxtr*⁺ cells in the dLS between male and female mice. ^****^P < 0.0001, unpaired *t t*est, *t*_(39)_ = 5.62, Cohen’s *d* = 1.76, 95% CI [147.4, 313.0]. *n* = 20-24 randomly selec*t*ed fields across 6–8 sections per mouse. *n* = 4 mice per group. **(D)** Schematic diagram of ACSF/TGOT injection into the dLS of C57BL/6 male mice. **(E)** Representative image showing the cannula placement. Scale bar = 200 μm. **(F)** Administration of TGOT significantly enhanced the preference for cued cinnamon in male mice during the STFP. Food consumption: ACSF: ^*^P = 0.049, paired *t t*est, *t*_(13)_ = 2.17, Cohen’s *d* = 0.58, 95% CI [–0.17, –0.00036]; TGOT:^****^P < 0.0001, paired *t* tes*t*, *t*_(13)_ = 6.38, *Cohen’s d* = –1.70, *95% CI* [–0.26, –0.13]. Preference index: *n.s.*, *P* = 0.064, unpaired *t t*es*t*, *t*_(26)_ = 1.94, Cohen’s *d* = 0.73, 95% CI [–1.21, 40.84]. **(G)** Administra*t*ion of TGOT significantly enhanced the preference for cued cumin in male mice during the STFP. Food consumption: ACSF: ACSF: ^*^P = 0.026, paired *t* tes*t*, *t*_(14)_ = 2.50, Cohen’s *d* = –0.65, 95% CI [–0.17, –0.013]; TGOT: ^****^P < 0.0001, paired *t t*est, *t*_(13)_ = 6.11, Cohen’s *d* = –1.63, 95% CI [–0.24, –0.12]. Preference index: ^*^P = 0.041, unpaired *t* tes*t*, *t*_(27)_ = 2.14, Cohen’s *d* = 0.80, 95% CI [1.031, 47.95]. *n* = 14 and mice for ACSF and TGOT group. Underlying data can be found in S1 Data.

To directly address whether dLS OXTR^+^ neuronal activity specifically reflect socially acquired food value, we conducted a new fiber photometry experiment using OXTR-promoter-driven GCaMP6s in the dLS (Fig 7A–7B). We recorded calcium activity during both innate preference and STFP tests on the same animals, using distinct odor pairs for each test. First, we recorded the real-time calcium activity during the innate food preference test (S12A Fig). Mice exhibited a robust innate preference for cocoa-flavored food (^**^P = 0.0027, paired *t* test, S12B Fig), but dLS OXTR-positive neurons showed no significant calcium responses during consumption of either cinnamon or cocoa food (AUC: *n.s.*, *P* = 0.84, paired *t* test; Peak ΔF/F: *n.s.*, *P* = 0.95, Wilcoxon matched-pairs signed rank test; Mean ΔF/F: *n.s.*, *P* = 0.84, paired *t* test, S12C–S12E Fig), indicating that these neurons do not simply encode innate food preference or feeding execution. During the STFP test, mice showed a significant preference for the socially cued food over the uncued food (^**^P = 0.0078, Wilcoxon matched-pairs signed rank test, Fig 7C). Notably, the same neuronal population showed robust calcium responses during social interaction (AUC of social interaction: ^**^P = 0.0063, paired *t* test, Fig 7D–7E). Moreover, their calcium signals were significantly greater during socially cued-food consumption than during uncued food consumption (AUC of food eating: ^**^P = 0.0044, paired *t* test; Peak ΔF/F: ^**^P = 0.0017, paired *t* test; Mean ΔF/F: ^**^P = 0.0044, paired *t* test, Fig 7D and 7F–7H). These results demonstrate that dLS OXTR-positive neurons differentiate between socially cued and uncued food, and do not simply reflect general preference, supporting a specific role for dLS OXTR signaling in encoding socially acquired food value.

**Fig 7:**
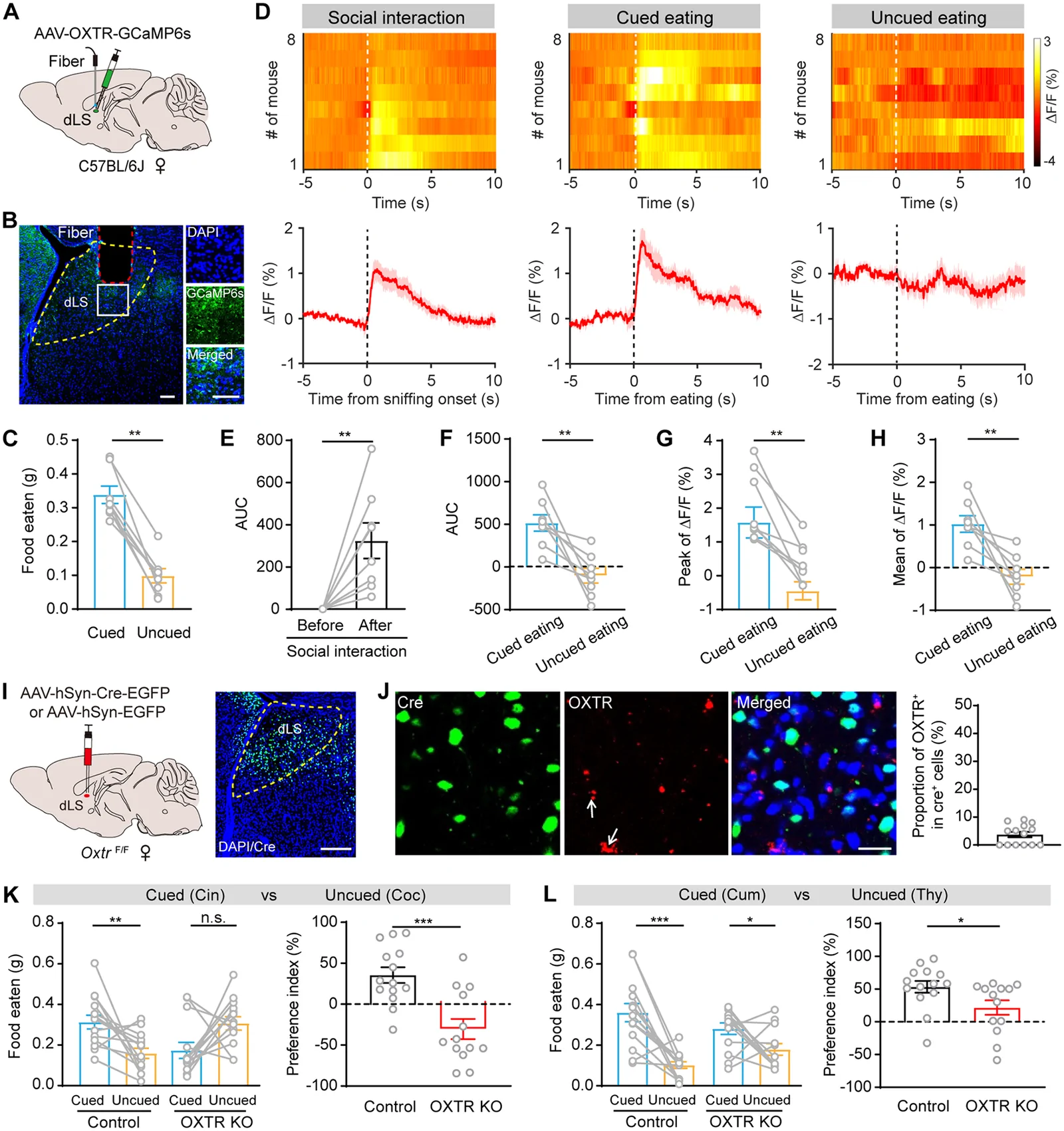
OXTR signaling in dLS neurons is necessary for STFP regulation. **(A)** AAV-OXTR-GCaMP6s was injected into the dLS of C57BL/6J mice for calcium signal recording. **(B)** A representative image showing GCaMP6s expression in dLS OXTR^+^ neurons and the fiber placement within the dLS. Scale bar = 100 μm. **(C)** Behavioral assessments of mice during calcium activity recordings. Cued vs. uncued: ^**^P = 0.0078, Wilcoxon matched-pairs signed rank test, *W* = –36, *r*_rb_ = –1.0, 95% CI [–0.39, –0.15]. *n* = 8 mice. **(D)** Heat maps (top) and averaged traces (bottom) illustrating the calcium responses during the three sessions. **(E)** Quantification of the AUC of the calcium signal in dLS OXTR^+^ neurons during social interaction. ^**^P = 0.0063, paired *t* test, *t*_(7)_ = 3.85, Cohen’s *d* = 1.36, 95% CI [123.7, 518.2]. **(F)** Quantification of the AUC of the calcium signal during food eating. ^**^P = 0.0044, paired *t* test, *t*_(7)_ = 1.46, Cohen’s *d* = –0.58, 95% CI [–972.0, –264.4]. **(G-H)** The mean and peak calcium response of OXTR^+^ neurons during cued and uncued food eating. Peak ΔF/F: ^**^P = 0.0017, paired *t* test, *t*_(7)_ = 4.92, Cohen’s *d* = –1.74, 95% CI [–2.40, –0.84]; Mean ΔF/F: ^**^P = 0.0044, paired *t* test, *t*_(7)_ = 4.13, Cohen’s *d* = –1.46, 95% CI [–1.95, –0.53], *n* = 8 mice. **(I)** Left: AAV-hSyn-Cre-EGFP or AAV-hSyn-EGFP injection into the dLS of *Oxtr*^F/F^ female mice. Right: representative images showing Cre-EGFP expression in the dLS. Scale bar = 200 μm. **(J)** Representative immunostaining image showing OXTR knockdown efficiency, with quantification count of OXTR^+^ cells within Cre^+^ population. Scale bar = 20 μm. **(K)** OXTR knockout abolished the preference for the cued cinnamon during the STFP. Food consumption: Control: ^**^P = 0.0065, paired *t* test, *t*_(13)_ = 3.24, Cohen’s *d* = –0.87, 95% CI [–0.26, –0.051]; OXTR KO: *n.s.*, *P* = 0.068, Wilcoxon matched-pairs signed rank test, *W* = 53, *r*_rb_ = 2.29, 95% CI [–0.076, 0.29]. Preference index: ^***^P = 0.0008, Mann Whitney test, *U* = 25, *r*_rb_ = 0.72, 95% CI [–103.3, –35.04]. **(L)** OXTR knockout abolished the preference for the cued cumin during the STFP. Food consumption: Control: ^***^P = 0.0002, paired *t* test, *t*_(13)_ = 5.23, Cohen’s *d* = –1.40, 95% CI [–0.36, –0.15]; OXTR KO: ^*^P = 0.048, Wilcoxon matched-pairs signed rank test, *W* = −57, *r*_rb_ = –0.63, 95% CI [–0.24, 0.09]. Preference index: ^*^P = 0.013, Mann Whitney test, *U* = 40, *r*_rb_ = 0.56, 95% CI [–57.03, –3.95]. *n* = 14 mice for each group. *n* = 14 mice for each group. Underlying data can be found in S1 Data.

To further establish the necessity of dLS-specific OXTR in dLS during STFP and to complement our pharmacological findings, we conditionally knocked out OXTR by injecting AAV-hSyn-Cre-EGFP into the dLS of *Oxtr*^flox/flox^ mice (Fig 7I–7J). Control mice displayed a robust preference for the cued food (Cin versus Coc: ^**^P = 0.0065, paired *t* test; Cum versus Thy: ^***^P = 0.0002, paired *t* test. Fig 7K and 7L Left), indicating intact STFP. In contrast, OXTR knockout significantly impaired this preference (Cin versus Coc: *n.s.*, *P* = 0.068, Wilcoxon matched-pairs signed rank test; Cum versus Thy: ^*^P = 0.048, Wilcoxon matched-pairs signed rank test. Fig 7K and 7L left), as reflected by lower preference indices compared to controls (Cin versus Coc: ^***^P = 0.0008, Mann Whitney test, *U* = 25; Cum versus Thy: ^*^P = 0.013, Mann Whitney test, Fig 7K–7L Right). Together, these results confirm that OXTR expression in dLS neurons is indispensable for STFP.

### Oxytocin increase the excitability of dLS neurons

To determine whether oxytocin signaling modulates STFP by regulating LS neuronal activity, we performed cell-attached recording in acute dLS slices (Fig 8A). After recording spontanous firing of dLS neurons, the oxytocin analogue TGOT (1 μM) was bath-applied. TGOT gradually increased the spontaneous firing frequency of LS neurons (^**^P = 0.0078, Wilcoxon matched-pairs signed rank test, Fig 8B–8E). To further examine the effect of TGOT on synaptic transmission, we recorded miniature inhibitory postsynaptic currents (mIPSC) and miniature excitatory postsynaptic currents (mEPSC) in LS neurons. As shown in Fig 8F, TGOT selectively increased mIPSC frequency without altering amplitude (Frequency: ^*^P = 0.047, paired *t* test; Amplitude: *n.s.*, *P* = 0.59, paired *t* test. Fig 8F). In contrast, TGOT application had no significant effect on mEPSC frequency or amplitude (Frequency: *n.s.*, *P* = 0.70, Wilcoxon matched-pairs signed rank test; Amplitude: *n.s.*, *P* = 0.27, paired *t* test. Fig 8G). Considering the predominance of GABAergic neurons and local inhibitory connectivity in the dLS [24], the increase in mIPSC frequency likely reflects enhanced presynaptic GABA release due to TGOT-induced excitation of local GABAergic neurons. Together, these results demonstrate that oxytocin receptor activation elevates GABAergic output from dLS neurons.

**Fig 8:**
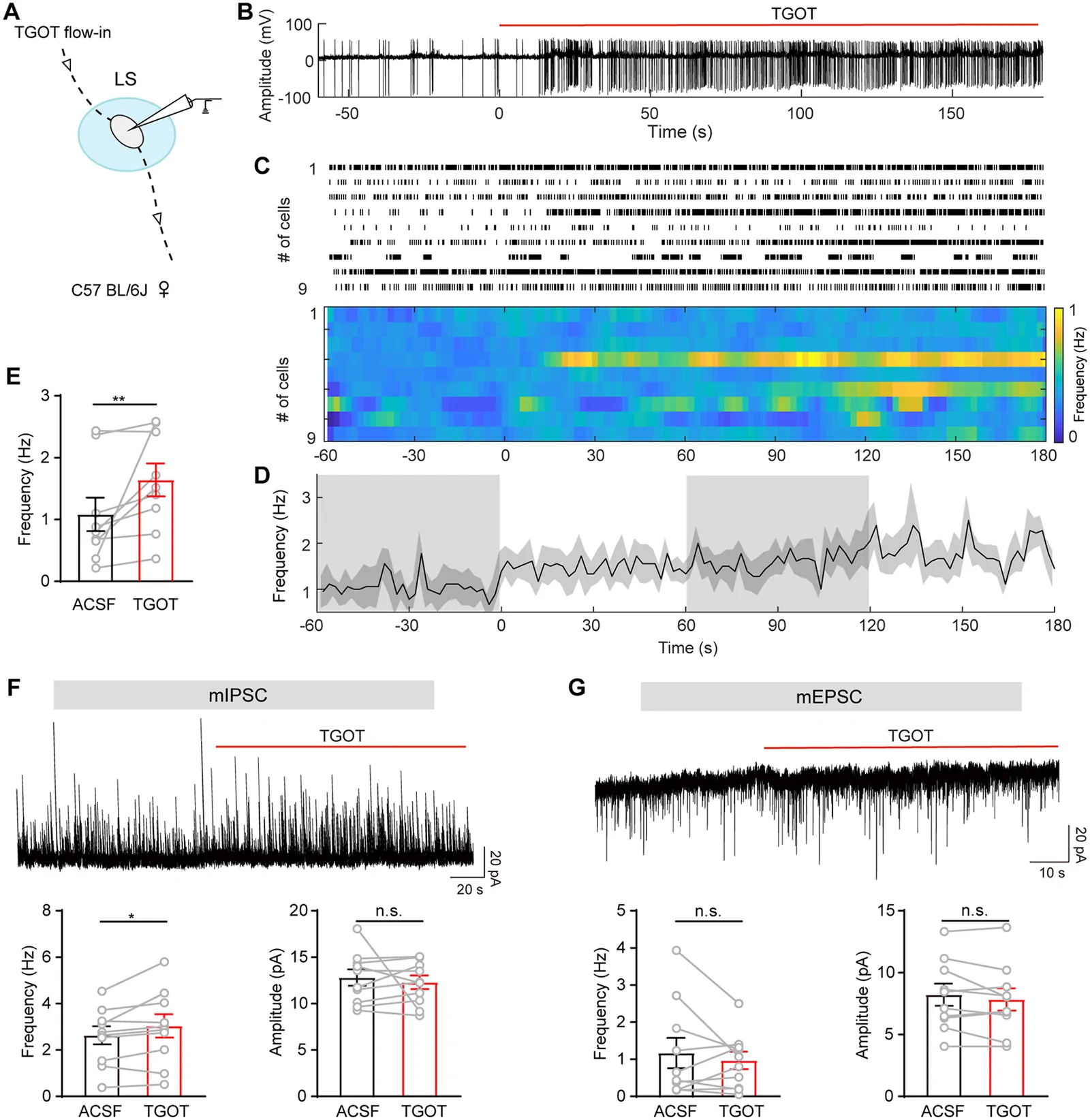
Oxytocin increase the excitability of LS neurons in vitro. **(A)** Schematic of the experimental paradigm. **(B–D)** Spontaneous firing activity of dLS neurons following TGOT application: representative action potential trace (B), raster plots and heatmaps (C), and averaged firing rate across cells (D). **(E)** Comparison of firing rate before and after TGOT perfusion across all cells recorded. ^**^P = 0.0078, Wilcoxon matched-pairs signed rank test, *W* = 43, *r*_rb_ = 0.96, 95% CI [0.083, 0.92]. **(F)** Up: the representive mIPSCs trace recorded before and after TGOT perfusion; Bottom: comparison of the frequency and amplitude of mIPSCs. Frequency: ^*^P = 0.047, paired *t* test, *t*_(9)_ = 2.30, Cohen’s *d* = 0.73, 95% CI [0.0065, 0.76]; Amplitude: *n.s.*, *P* = 0.59, paired *t* test, *t*_(9)_ =0.57, Cohen’s *d* = –0.18, 95% CI [–2.79, 1.67]. **(G)** Up: the representive mEPSCs trace recorded before and after TGOT perfusion; Bottom: comparison of the frequency and amplitude of mEPSCs. Frequency: *n.s.*, *P* = 0.70, Wilcoxon matched-pairs signed rank test, *W* = –9, *r*_rb_ = –0.16, 95% CI [–1.43, 0.63]; Amplitude: *n.s.*, *P* = 0.21, paired *t* test, *t*_(9)_ =1.36, Cohen’s *d* = –0.43, 95% CI [–1.004, 0.25]. *n* = 10 cells from 5 mice. Underlying data can be found in S1 Data.

Having identified the effect of oxytocin on LS neurons, we next investigated the underlying signaling pathways. Oxytocin receptors are predominantly coupled to Gq proteins, which activate phospholipase C (PLC) to mobilize calcium from intracellular stores [30]. To assess the contribution of PLC signaling to the effect of oxytocin, we applied the selective PLC inhibitor U73122 (S13A Fig). In the presence of 10 μM U73122, TGOT no longer significantly increased the spontaneous firing frequency of dLS neurons (*n.s.*, *P* = 0.85, paired *t* test, S13B–S13E Fig). We then asked whether KCNQ potassium channels, which typically constrain neuronal excitability downstream of Gq-PLC signaling, modulate the oxytocin-induced response in dLS neurons. Pretreatment with the KCNQ inhibitor XE991 (1 μM) did not block the excitatory effect of TGOT (^*^P = 0.015, paired *t* test, S13F–S13J Fig), indicating the OXTR-PLC pathway enhances LS neuronal excitability independently of KCNQ channel suppression. Together, these results demonstrate that OXTR-PLC pathway elevates LS neuronal activity through a non K^+^-dependent mechanism.

## Discussion

Here, we demonstrate that the LS modulates social learning and shapes food preference in a subregion-specific manner. During STFP, both calcium transients and neuronal activity in the LS increased following social learning and reliably distinguished cued from uncued food. Inhibition of dLS neurons abolished cued food preference, whereas vLS inhibition had no effect, highlighting a selective and essential role of the dLS in STFP. OXTR signaling is necessary for STFP and can facilitate STFP formation in male mice. Mechanistically, we found that oxytocin enhances neuronal excitability via OXTR-mediated activation of the PLC pathway.

Mammals continuously integrate olfactory cues from food and their surroundings within changing environmental and physiological contexts to guide decision-making. Previous studies have shown that STFP engages a distributed circuit involving olfactory regions such as the olfactory epithelium, olfactory bulb, anterior olfactory nucleus; olfactory memory-related structures like the posteromedial nucleus of the cortical amygdala; and reward-processing centers, notably the medial prefrontal cortex and the nucleus accumbens [2,3,31]. However, how olfactory inputs are consolidated into olfactory memory and ultimately shape food preference remains unknown. Contextual odor information is relayed indirectly via the hippocampus [5], a hub critical for encoding, storing, and retrieving social memories [10,32]. The LS, a region known to be involved in both social memory and feeding motivation [6,12,13,33], has been reported to replay hippocampal output to subcortical structures [12]. Given its unique position at the intersection of memory and motivation, the LS emerges as a compelling candidate for linking hippocampal social representations to the adaptive control of feeding behavior. Growing evidence suggests that hippocampal projections to the LS regulate social behaviors [12–14], indicating that hippocampus may integrate olfactory and social learning information and relay it to the LS during STFP. In line with this, we observed increased calcium transients and spike activity in the LS during social learning, and chemogenetic inhibition of LS neurons specifically impaired subsequent cued-food preference. Nevertheless, whether and how the projections between the hippocampus and LS mediate social memory in STFP awaits further experimental elucidation.

The LS acts as a pivotal neural hub in the regulation of feeding behavior [34]. Chemogenetic inhibition of LS neurons increased food intake, while their activation suppresses it [35,36]. Our data indicate that neither inhibition nor activation of LS neurons significantly alters total food intake during STFP (S7 Fig). This discrepancy may stem from the specific behavioral context of STFP, where social and cognitive factors likely modulate feeding regulation in ways that override the direct homeostatic control mediated by LS neurons. Our data reveal that LS neuronal activity, as reflected by calcium dynamics and firing activity, discriminates between socially cued and uncued foods, further supporting its role in feeding regulation. Thus, the LS appears to transform socially derived signals into selective changes in food preference, likely by modulating feeding motivation before engaging downstream consumption circuits. This positions the LS as a critical interface where social information integrates with motivational drives to guide adaptive feeding decisions. Recent studies indicates that the lateral hypothalamic area (LHA) serves as a key downstream target of the LS in feeding control: inhibitory LS to LHA projections participate in context-conditioned feeding [33,37], while gamma-rhythmic LS input to the LHA promote food approach [38]. Importantly, LHA neurons themselves modulate responses to sweet and bitter tastes [39], positioning the LHA as a node capable of guiding food preference. These findings suggest that motivation-related signals from the LS may engage LHA circuit to shape food preference, particularly in socially modulated contexts such as STFP. Nevertheless, whether the LS–LHA circuit specifically translates feeding motivation into particular food preferences remains untested, and we acknowledge this as a key limitation of the current study; future experiments using pathway-selective manipulations will be required to directly establish the necessity and sufficiency of this circuit in STFP.

The present study establishes that oxytocin signaling is indispensable for STFP, as demonstrated through integrated pharmacological, anatomical, and behavioral evidence. The sex-specific expression pattern of OXTR in the dLS likely underlies the observed behavioral dimorphism between males and females, though whether oxytocin release within the dLS during STFP itself differs by sexes remains to be determined. Endogenous oxytocin is primarily synthesized in the paraventricular (PVN) and supraoptic nuclei of the hypothalamus. A distinct subpopulation of small-vesicle PVN neurons projects oxytocinergic fibers wildly to limbic and cortical regions, including the LS [40,41]. Consistent with its role in social processing, oxytocin enhances social interaction and facilitates social learning across various contexts [13,28]. Notably, oxytocin signaling within the LS has been directly implicated in modulating social learning [42–44].

Although OXTRs can couple to diverse G-protein to elicit distinct physiological outcomes [45], their downstream signaling within LS neurons remains poorly characterized. Here, we demonstrate that exogenous oxytocin enhances dLS neuronal excitability, consistent with a previous report [24]. Mechanistically, we establish that OXTR activation in dLS neurons recruits the PLC signaling cascade to drive increased excitability. While potassium channels underlying the M-current (composed of KCNQ subunits) are often modulated by GPCR signaling to regulate somatic excitability [46,47], the KCNQ inhibitor XE991 did not attenuate the excitatory effect of the OXTR agonist TGOT in dLS neurons. This indicates that oxytocin-induced excitation in the dLS operates independently of KCNQ mediated K^+^ conductance, pointing to a distinct downstream effector pathway.

It should be noted that the evidence supporting a requirement of dLS OXTR signaling for STFP is primarily derived from experiments conducted in female mice. Although dLS OXTR neurons in males also encode socially acquired food value, and pharmacological enhancement of OXTR signaling in the dLS facilitates STFP performance in males, the necessity of dLS OXTR for STFP in males, as well as the causal relationship between OXTR expression levels and behavioral performance in either sex, remains to be determined. In addition, while atosiban has been traditionally used as an OXTR antagonist, it has recently been identified as a Gi-biased agonist rather than a neutral receptor blocker [26]. Notably, this Gi agonist is consistent with our chemogenetic Gi activation results, both of which suggest that inactivation of dLS neurons impairs STFP.

Collectively, our study demonstrates functional divergence across LS subregions in STFP. Neuronal activity in the dLS encodes the social value of food and is necessary for STFP. The observed sex differences in STFP are likely arise from differential OXTR expression within the dLS. Furthermore, we establish that oxytocin enhances dLS neuronal excitability via activation of the PLC signaling pathway, thereby defining a specific molecular mechanism through which social neuropeptidergic signaling shapes adaptive feeding behavior.

## Materials and methods

### Ethics statement

The experimental protocols in this study were approved by the Institutional Animal Care and Use Committee (IACUC) of Xuzhou Medical University under the Regulations for the Administration of Affairs Concerning Experimental Animals (1988) in China. The approval number is 202308T005. The use and care of animals were performed in the animal facility of Xuzhou Medical University managed by the Office of Laboratory Animal Resources of Xuzhou Medical University.

### Animals

Male and female C57BL/6J mice (Gempharmatech Co., Ltd., Nanjing, China) and *Oxtr*^flox/flox^ mice (Jax 008471, Maine, USA), aged 8–14 weeks, were utilized for the experiments. Mice were housed under controlled conditions, adhering to a 12-hour light/dark cycle, with ambient temperature maintained at 22–26 °C and relative humidity between 40%–70%.

### Stereotaxic surgeries

All viral vectors, including AAV-hSyn-GCaMP6s, AAV-OXTR-GCaMP6s-EGFP, AAV-AAV-hSyn-hM4Di-mCherry, AAV-hSyn-hM3Dq-mCherry, AAV-hSyn-mCherry, AAV-hSyn-GtACR1-EGFP AAV-hSyn-Cre-EGFP, and AAV-hSyn-EGFP were purchased from BrainVTA (Wuhan, China). For precise viral administration, animals were anesthetized with pentobarbital sodium (90 mg/kg, i.p.) and positioned in a stereotaxic apparatus (RWD Life Science, Shenzhen, China). A regulated heating pad maintained the body temperature. Stereotaxic coordinates for virus injections was (in mm): AP 0.6, ML 0.3, DV 3.5 (LS), AP 0.6, ML 0.3, DV 3.2 (dLS), AP 0.6, ML 0.45, DV3.85 (vLS); for optical fiber implantation is AP 0.6, ML 0.3, DV 3.0 (dLS). A craniotomy was performed at the designated coordinates, followed by the injection of the virus (200 nL) using a glass pipette and microsyringe pump (Stoelting Quintessential Injector, Stoelting, USA) at a rate of 20 nL/min. The pipette was held in place for an additional 5-min post-injection, then retracted 0.5 mm and, after a 10-minute interval, gradually removed from the brain tissue. After the virus injection, for fiber photometry and in vivo optogenetic inhibition, optical fiber (O.D. 200 μm; NA 0.37; length 5.0 mm; Newdoon, Hangzhou, China) were implanted in the dLS and vLS. For cannula infusion of drugs, cannulas (RWD Life Science, Shenzhen, China) were implanted into the i.c.v. (AP 0.1 mm, ML 0.85 mm, DV 2.5 mm). The optical fiber and cannula were fixed in place with dental acrylic. Mice were housed individually for at least seven days after surgery for recovery.

### Histology

To verify the expression of c-Fos and OXTR, the site of infection, and the position of fiber or cannula placement, we prepared frozen brain sections. Mice were deeply anesthetized with pentobarbital sodium and subsequently perfused intracardially with 0.9% saline followed by 4% paraformaldehyde (PFA) in 0.1 M phosphate buffer solution (PBS, pH 7.4). The brains were then extracted, post-fixed in 4% PFA at 4 °C overnight, and cryoprotected in 30% sucrose (w/v) in PBS until they sank. Coronal brain sections (30 μm) were obtained using a freezing microtome (CM1860, Leica, Wetzlar, Germany) and mounted on glass slides. For immunostaining, sections were washed three times in PBS and blocked in 10% goat serum with 0.2% Triton X-100 for 1 hour at room temperature. They were then incubated with c-Fos (2250s, Cell Signaling Technology, Danvers, MA, USA), and OXTR (23045-1-AP, proteintech) primary antibody diluted in the blocking buffer overnight at 4 °C. Following three PBS washes, sections were incubated with an Alexa Fluor 594-conjugated secondary antibody (A11037, Carlsbad, CA, USA) for 1 hour at room temperature. Nuclei were counterstained with 4′,6-diamidino-2-phenylindole (DAPI) for 10 min at room temperature. After six PBS washes, sections were cover-slipped with mounting medium and imaged using a confocal microscope (Zeiss, Oberkochen, Germany), then the images were processed with ZEN 2011 software(Zeiss). For histological quantification, the brains were coronally sectioned (20 μm) and nonconsecutive sections were randomly collected for c‑Fos or OXTR immunostaining. The number of sections and mice used for quantification were provided in the figure legends. All image collection and data analysis were performed by experimenters blinded to the experimental conditions.

### Oxtr RNAscope

Brain tissues from two‑month‑old female and male mice (*n* = 3 per sex) were cryosectioned at 20 μm thickness and mounted onto SuperFrost Plus glass slides. In situ hybridization for mouse *Oxtr* mRNA was performed using the RNAscope v2 Assay kit (cat. no. 323100; Advanced Cell Diagnostics, USA) with the Mm‑*Oxtr* probe (cat. no. 412171), following the manufacturer’s instructions for chromogenic detection, as previously described [27]. Briefly, slides were baked at 40 °C for 1 h, gradient dehydrated with ethanol, and incubated with hydrogen peroxide for 10 min at room temperature. Antigen retrieval was carried out with Target Retrieval Reagent for 5 min at 100 °C, followed by protease digestion with Protease III for 30 min at 40 °C. Probe hybridization and signal amplification were performed according to the manufacture’s protocol. Images were acquired using a confocal microscope (Zeiss, Oberkochen, Germany)at ×40 magnification and processed with ZEN 2011 software (Zeiss). The number of sections and brains used for quantification were provided in the figure legends.

### *In vivo* electrophysiological recordings

#### Microelectrode implantation.

Under pentobarbital sodium-induced anesthesia, confirmed by toe pinch, mice were maintained at 37 °C using a heating blanket. They were positioned in a stereotaxic frame, and eye ointment was applied. The scalp fur was removed from the midline of the orbits to the midpoint between the ears. A small craniotomy was performed above the dLS for microelectrode implantation (4 × 4-channel microwire array electrode, Kedou Brain-computer Technology, Suzhou, China). Reference electrodes were placed 0.6 mm posterior to bregma and 0.3 mm lateral to the midline. The microelectrodes were inserted to a depth of 3.2 mm, with recordings made during implantation to ensure optimal placement within the LS. Signals were transmitted to an electrophysiological acquisition system (NeuroLego, Jiangsu Brain Medical Technology, Nantong, China) through a headstage and a 16-channel amplifier (bandpass filtered at 1–5,000 Hz, 2,000 × gain). Spike signals were filtered at 300–5,000 Hz and sampled at 40 kHz, while LFPs were filtered at 1–300 Hz and sampled at 1 kHz. The microelectrodes were secured with dental acrylic, and recordings commenced after a minimum 10-day recovery period.

#### Off-line spike sorting and unit data statistics.

For *in vivo* electrophysiological recordings, the primary unit of analysis was the signal unit isolated from the raw multi-channel signals. Spikes were sorted from raw data using Offline Sorter V4 software (Plexon, Dallas, USA), with unit separation achieved through principal component analysis. Units with less than 0.75% of interspike intervals under 1 ms were classified as single units, resulting in unimodal firing rate distributions. Data from 2 seconds before to 4 s after the onset of social interaction or food intake were analyzed, with mean firing rate (MFR) traces generated by averaging spike rates in 100-ms bins. Baseline spontaneous firing rates were calculated from the 2-s pre-interaction or pre-feeding period, while evoked firing rates were averaged across the 2-s post-interaction or post-feeding period. A permutation test compared baseline and evoked firing rates to determine response significance. Normalized MFR was calculated as (MFR–MFR of baseline)/MFR of baseline.

#### Off-line analysis of LFP signals.

For LFP signals analysis, raw data 2 s prior to the onset of odor stimulation were selected as representing ongoing LFP activity. As in our previous studies [48], raw LFP signals were divided into 4 frequency bands: theta (2–12 Hz), beta (15–35 Hz), low-gamma (36–65 Hz), and high-gamma (66–95 Hz). For analysis of LFP responses, windows 2 s prior to and 2 s after the onset of interaction or feeding were selected. For each trial, the signal was normalized to a baseline value of 1. All trials corresponding to social interaction, cued-food eating, or uncued-food eating were then averaged based on the normalized data.

### Fiber photometry

Fiber photometry was performed using a previously described system [48,49]. Fluorescent signals were recorded using a 488-nm laser beam, reflected by a dichroic mirror, focused by an objective lens, and transmitted via an optical commutator. GCaMP6s fluorescence was collected, bandpass filtered, and detected by a photomultiplier tube. The resulting voltage signal was low-pass filtered and digitized at 500 Hz. The test mice were habituated to the fiber optic patch cord for 10 min on three consecutive days. Behavioral assays were video-recorded alongside GCaMP6s signal acquisition during social interaction tests and feeding. Nose-to-nose contact and anogenital sniffing were defined as social interaction events for fiber photometry analysis. Fluorescence change (ΔF/F) was calculated as (F − F_0_) / F_0_, with F_0_ representing baseline fluorescence averaged over a 5-s-long control time window, which preceded the onset of social interaction or feeding. ΔF/F values were visualized as heat maps or trial-averaged traces. The area under the curve (AUC), Peak and Mean ΔF/F were calculated over a 5-s window following the onset of social interaction, cued-food eating, or uncued-food eating.

### Intraperitoneal injection and cannula infusion of drugs

For cannula infusion of drugs, the stereotaxic coordinate for i.c.v. injections were (in mm): AP 0.1, ML 0.85, DV 2.5; for dLS injections were(in mm): AP 0.6, ML 0.3, DV 3.2. For intraperitoneal administration, Clozapine N-oxide (CNO, 3.33 mg/kg, BrainVTA) was administered 45 min prior to behavioral assays. Atosiban (0.02 mM, Sigma-Aldrich, A3480), V1aR-antagonist (0.03 mM, Sigma, S5701), and dOVT (0.5 mM, MCE, HY-P4895) were delivered bilaterally into the target region via implanted cannulae at a volume of 1 μL per side and an infusion rate of 200 nl/min, beginning 15 min before behavioral testing. The injector cannula remained in situ for an additional 2-min post-injection to facilitate drug diffusion.

### Optogenetic manipulation

Optogenetic activation of GtACR1 was achieved using a 473-nm laser generator (Newdoon, Hangzhou, China). For the inhibition of GtACR1-expressing neurons, light was transmitted via a patch cable to the optical fiber implanted in the LS. Laser intensity was set to approximately 6 mW for somatic activation. Light stimulation was delivered throughout the social interaction period of STFP, or during the full duration of the three-chamber test. To avoid heating effects on the tissue from sustained light, we applied 2-s episodes of 20 Hz blue light (5-ms pulses) with 1-s inter-episode intervals.

### In vitro electrophysiological recordings

#### Brain slice preparation.

Mice were anesthetized with pentobarbital sodium (90 mg/kg, i.p.) and perfused intracardially with an ice-cold dissection buffer saturated with 95% O_2_/5% CO_2_, comprising (in mM): 234 sucrose, 11 glucose, 24 NaHCO_3_, 2.5 KCl, 1.25 NaH_2_PO_4_, 0.5 CaCl_2_, and 2 MgSO_4_. Brains were extracted and sectioned coronally (250 µm thick) on a vibratome (VT 1200S, Leica, Wetzlar, Germany) to obtain slices containing the LS. Slices were then incubated in oxygenated artificial CSF (ACSF, [in mM]: 126 NaCl, 26 NaHCO_3_, 2.5 KCl, 1.25 NaH_2_PO_4_, 2 CaCl_2_, 2 MgCl_2_, and 10 glucose) at 37 °C for 60 min before being maintained at room temperature for electrophysiological recordings.

#### Patch-clamp recordings.

Slices were placed in a recording chamber and continuously perfused with carbogen-saturated ACSF at 2–3 ml/min. Neurons were visualized with an upright microscope (FN-1, Nikon, Tokyo, Japan) using a 60× water-immersion lens and wide-field fluorescence. Recordings were made with a Multiclamp 700B amplifier and a Digidata 1440A A/D converter (Molecular Devices, San Jose, USA).

Cell-attached and whole-cell recordings were performed as previously described [38]. Slices were perfused with ACSF supplemented with 1 μM TGOT to mimic the effect of oxytocin, 10 μM U73122 to block PLC pathway, and 1 μM XE991 to block KCNQ.

To record miniature inhibitory postsynaptic currents (mIPSCs), neurons were held at 0 mV and recording electrodes were filled with a high-Cl internal solution containing (in mM): 125 cesium methane sulfonate, 7 CsCl, 10 Hepes, 4 MgATP, 0.3 Na_3_GTP, 0.5 EGTA, 10 Na_2_-phosphocreatine and 5 QX-314 (300 mOsm, pH7.3 adjusted with CsOH). TTX (1 μM), CNQX (10 μM), and DL-2-amino-5-phosphonopentanoic acid (DL-APV) (50 μM) were added to block voltage-gated sodium channels, AMPA receptors and NMDA receptors, respectively. For whole-cell recordings of action potentials and miniature inhibitory postsynaptic currents (mEPSCs). The recording electrodes were filled with a K^+^ internal solution containing (in mM): 135 potassium gluconate, 5 KCl, 0.5 CaCl_2_, 10 Hepes, 2 MgATP, 0.1 Na_3_GTP and 5 EGTA (300 mOsm, pH 7.3 adjusted with KOH). To isolate mEPSCs, neurons were held at −70 mV, and the recording was performed in the presence of TTX (1 μM) and the GABAA receptor antagonist bicuculline (10 μM) in the ACSF.

### Behavioral assessments

#### Innate food preference test.

To determine innate food preferences, male and female mice were simultaneously presented with two pairs of scented food: cinnamon versus cocoa, and cumin versus thyme. These odor pairs were selected based on previous studies showing that mice exhibit an innate preference for cocoa and thyme over cinnamon and cumin [1,6,50], allowing us to test whether social experience can override this innate preference. The innate food preference test was conducted as previously described [1–3]. Food pellet with cinnamon (1%), cocoa (2%), cumin (0.75%), or thyme (0.5%) were baked in an oven (55 °C, 2 hours) until the food cookies are completely dry. Three days before test, mice were placed into a chamber with two dishes containing plain food, where they were habituated to eating food pellet from dishes. On the day of innate food preference training, the mice naive to the odors were fasted for 15–18 hours and then were moved to the same cage with two dishes containing food with cinnamon/cocoa or cumin/thyme. The positioning of the food dishes was randomly selected to avoid position preference of the test mice. The mice were allowed to eat the different food for 60 min before being returned to their home cages. The consumed food was measured and their ratios to total food consumption were calculated.

#### Social transmission of food preference (STFP) test.

STFP test was performed according to established protocols [1–3,6]. The behavior test was evaluated using a three-chamber apparatus (72 cm length × 23 cm width × 25 cm height) with transparent acrylic partitions and two dishes (10 cm in diameter) positioned in the lateral chambers. Three days before test, both demonstrator and observer mice were placed into a chamber with two dishes containing plain food, where they were habituated to consuming food pellets from the dishes. Before the STFP test, all mice were singly housed in fresh cages and food-deprived for 15–18 h. On the training day, the demonstrator mice were allowed to eat cued (1% cinnamon or 0.5% cumin-scented) food for 30 min, and were then put into the middle chamber and allowed to interact with the observer mice for 30 min. Observer mice were sex- and strain-matched with demonstrators, two weeks older to prevent fighting. They were neither littermates nor had any prior contact with the demonstrator mice. All mice were at least 8 weeks old upon testing. To minimize the potential impact of estrous cycle on STFP performance, female mice were randomly assigned across experimental groups to ensure balanced representation. After social interaction, the demonstrator mice were taken away, the observer mice were allowed freely explore two dishes containing cued (cinnamon or cumin-scented) and uncued (cocoa or thyme-scented) food for 60 min within the chamber. Food consumption was quantified for each scent, and a preference index was calculated as: (cued food intake – uncued food intake)/(cued food intake + uncued food intake) × 100%, modified from a previous study [6]. For fiber photometry or in vivo electrophysiological recordings, observer mice were given a 30-min free exploration period.

Behavioral metrics were captured and quantified using EthoVision XT software (Noldus Information Technology b.v., Wageningen, the Netherlands).

#### Three-chamber social interaction test.

The three-chamber test was conducted as previously described [49]. We ensured that the test mice were comfortable with being connected the patch cords to the implanted fibers via the ceramic sleeves. All mice were group-housed before testing to avoid isolation stress induced by single housing. The mice used as the social stimuli were sex- and strain-matched, and were 2 weeks younger than the test mice to prevent fighting. These stimulus mice were neither littermates nor had any prior contact with the test mice. Social behavior was evaluated using a three-chamber apparatus (72 cm length ×23 cm width ×25 cm height) with transparent acrylic partitions and two wire enclosures (10 cm in diameter) positioned in the lateral chambers. Two identical 250 ml plastic bottles were placed upright on top of each enclosure to prevent the test mouse from climbing the enclosure. The lighting for the three-chamber apparatus was maintained at <50 lux, ensuring even illumination. The test mice were habituated in the behavioral room for at least 1 hour.

Acclimatization of mice to the three-chamber occurred over a period of 10 min daily across 3 days. On the assessment day, test mice were initially placed in the central chamber for 10 min, followed by a 10-min exploration period of the entire apparatus, including the enclosures. Sociability was assessed by introducing a stimulus mouse beneath one enclosure, allowing the test mouse 10 min of free interaction (sociability assay). After a 10-min interval, the test mice were allowed to interact with the previously encountered stimulus mouse (now familiar and placed on the opposite side of the apparatus) and a novel stimulus mouse for 10 min (social novelty assay). After each assay, the apparatus, enclosures, and bottles were thoroughly cleaned with 75% ethanol to remove any residual odors that may affect subsequent tests. Each stimulus mouse interacted with a maximum of 5 test mice, and was discarded if it exhibited excessively grooming. Social engagement was measured by the cumulative duration of test mouse nasal contact within a 4 cm radius of the enclosure perimeter. The sociability discrimination index was computed as: [(time spent exploring the stimulus mouse – time spent exploring the empty cup)/(time spent exploring the stimulus mouse + time spent exploring the empty cup)] × 100%. The social novelty discrimination index was similarly calculated. Manual counting was performed by a researcher blinded to the animal treatment when the test mouse climbed the enclosures.

### Quantification and statistical analysis

Statistical analyses were conducted using GraphPad Prism or MATLAB software. Figures were prepared with Adobe Illustrator 2020. Data analysis and presentation complied with a previously published guideline [51]. The Gaussian distribution of the data was ascertained using the Anderson–Darling test. In cases where data followed a normal distribution, significance was determined using either a two-tailed paired or unpaired *t* test. Conversely, for nonnormally distributed data, the Wilcoxon signed-rank test or the Mann–Whitney test was applied. All summarized data are expressed as the mean ± standard error of the mean (SEM). Significance levels were established at ^*^P < 0.05, ^**^P < 0.01, ^***^P < 0.001, ^****^P < 0.0001, *n.s.* denoting not significant. Source data in all figures are provided in an excel data file named S1 Data submitted with this paper.

## Supporting information

S1 Fig STFP test does not influence the total food consumption in both sexes. **(A)** The total food consumption of two food pairs in male mice during the innate food preference test and STFP test. Left: Cin versus Coc: *n.s.*, *P* = 0.45, Mann Whitney test, *U* = 67.5, *r*_rb_ = 0.18, 95% CI [–0.068, 0.14], *n* = 11 mice for innate preference test, *n* = 15 mice for STFP test; Cum versus Thy: *n.s.*, *P* = 0.25, unpaired *t* test, *t*_(23)_ = 1.17, Cohen’s *d* = 0.48, 95% CI [–0.069, 0.25]; *n* = 10 mice for innate preference test, *n* = 15 mice for STFP test. **(B)** The total food consumption of two food pairs in female mice during the innate food preference test and STFP test. Left: Cin versus Coc: *n.s.*, *P* = 0.96, unpaired *t* test, *t*_(21)_ = 0.051, Cohen’s *d* = 0.022, 95% CI [–0.12, 0.17], *n* = 10 mice for innate preference test, n = 13 mice for STFP test; Cum versus Thy: *n.s.*, *P* = 0.58, unpaired *t* test, *t*_(29)_ = 0.55, Cohen’s *d* = 0.20, 95% CI [–0.061, 0.11]; *n* = 18 mice for innate preference test, *n* = 13 mice for STFP test. Underlying data can be found in S1 Data.
(TIF)

S2 Fig STFP is not maintained at 24 hours post‑training in either sex. **(A)** Summary plot of food consumption and preference index at 24 hour after STFP test in male mice. Food consumption: 0 hour: ^*^P = 0.043, paired *t* test, *t*_(11)_ = 2.30, Cohen’s *d* = –0.66, 95% CI [–0.16, –0.0032]; 24 hour: *n.s.*, *P* = 0.29, paired *t* test, t_(11)_ = 1.10, Cohen’s *d* = 0.32, 95% CI [–0.10, 0.31]. Preference index: ^*^P = 0.041, paired *t* test, *t*_(11)_ = 2.31, Cohen’s *d* = –0.67, 95% CI [–92.43, –2.19]. **(B)** Summary plot of food consumption and preference index at 24 hour after STFP test in female mice. Food consumption: 0 hour: ^***^P = 0.0009, paired *t* test, *t*_(11)_ = 4.49, Cohen’s *d* = –1.30, 95% CI [–0.39, –0.13]; 24 hour: ^**^P = 0.0056, paired *t* test, *t*_(11)_ = 3.44, Cohen’s *d* = 0.99, 95% CI [–0.053, 0.24]. Preference index: ^****^P < 0.0001, paired *t* test, *t*_(11)_ = 6.18, Cohen’s *d* = –1.78, 95% CI [–118.2, –56.09]. *n* = 12 mice for each group. Underlying data can be found in S1 Data.
(TIF)

S3 Fig The calcium activity of LS remains stable throughout the session during both cued and uncued food consumption. **(A, B)** Heat maps (top) and averaged traces (bottom) illustrating the calcium responses during nose-to-nose contact and anogenital sniff. **(C)** Quantification of the AUC of the calcium signal during nose-to-nose contact. ^***^P = 0.0005, Wilcoxon matched-pairs signed rank test, *W* = 78, *r*_rb_ = 1, 95% CI [74.69, 568.5]. **(D)** Quantification of the AUC of the calcium signal during anogenital sniff. ^**^P = 0.0034, Wilcoxon matched-pairs signed rank test, *W* = 70, *r*_rb_ = 0.90, 95% CI [56.18, 650.4]. **(E)** Quantification of the AUC of the calcium signal between nose-to-nose contact and anogenital sniff. *n.s.*, *P* = 0.89, Mann Whitney test, *U* = 69, *r*_rb_ = 0.042, 95% CI [–251.3, 274.3]. **(F, G)** All traces showing calcium responses during cued and uncued eating from a representative mouse. **(H)** Heat maps (top) and averaged traces (bottom) illustrating the calcium responses during cued food eating. **(I, J)** Peak and mean calcium responses of LS neurons, comparing the first and last trials of cued food eating. Peak ΔF/F: *n.s.*, *P* = 0.47, Wilcoxon matched-pairs signed rank test, *W* = –20, *r*_rb_ = –0.26, 95% CI [–1.75, 0.60]; Mean ΔF/F: *n.s.*, *P* = 0.57, Wilcoxon matched-pairs signed rank test, *W* = –16, *r*_rb_ = –0.21, 95% CI [–1.42, 0.69]. *n* = 12 mice. **(K)** Heat maps (top) and averaged traces (bottom) illustrating the calcium responses during uncued food eating. **(L, M)** Peak and mean calcium responses of LS neurons, comparing the first and last trials of uncued food eating. Peak ΔF/F: *n.s.*, *P* = 0.85, Wilcoxon matched-pairs signed rank test, *W* = 6, *r*_rb_ = 0.077, 95% CI [–1.14, 1.64]; Mean ΔF/F: *n.s.*, *P* = 0.85, Wilcoxon matched-pairs signed rank test, *W* = –6, *r*_rb_ = –0.077, 95% CI [–1.074, 0.68]. *n* = 12 mice. Underlying data can be found in S1 Data.
(TIF)

S4 Fig The calcium activity of LS neurons in male mice discriminates cued from uncued food during the STFP. **(A)** AAV-hSyn-GCaMP6s was injected into the LS of C57BL/6J male mice for calcium signal recording. **(B)** A representative image showing GCaMP6s expression and the fiber placement within the LS. Scale bar = 100 μm. **(C)** Behavioral assessments of mice during calcium activity recordings. ^*^P = 0.011, paired *t* test, *t*_(8)_ = 3.27, Cohen’s *d* = –1.09, 95% CI [–0.055, –0.32]. **(D)** Heat maps (top) and averaged traces (bottom) illustrating the calcium responses during the three sessions. **(E)** Quantification of the AUC of the calcium signal during social interaction. ^**^P = 0.0097, paired *t* test, *t*_(8)_ = 3.38, Cohen’s *d* = –1.088, 95% CI [164.4, 874.6]. **(F)** Quantification of the AUC of the calcium signal during cued and uncued food eating. ^*^P = 0.027, paired *t* test, *t*_(8)_ = 2.69, Cohen’s *d* = –0.90, 95% CI [–1,678, –129.7]. **(G-H)** The mean and peak calcium response of LS neurons during cued and uncued food eating. Peak ΔF/F: ^*^P = 0.029, paired *t* test, *t*_(8)_ = 2.65, Cohen’s *d* = –0.88, 95% CI [–3.69, –0.26]; Mean ΔF/F: ^*^P = 0.027, paired *t* test, *t*_(8)_ = 2.69, Cohen’s *d* = –0.90, 95% CI [–3.36, –0.26]. *n* = 9 mice. Underlying data can be found in S1 Data.
(TIF)

S5 Fig LS neurons exhibit distinct firing activity that selectively encode cued versus uncued food. **(A)** Schematic of the experimental paradigm. **(B)** Behavioral assessments of mice during in vivo electrophysiological recordings. Cued versus uncued: ^*^P = 0.033, paired *t* test, *t*_(8)_ = 2.58, Cohen’s *d* = –0.86, 95% CI [–0.11, –0.0063]. **(C)** Left: the representative raw spike trace of LS neurons. Right: example of principal-component analysis (PCA) used for clustering extracellular voltage recordings from microelectrodes, resulting in the identification of two distinct neuronal units (unit a in red, unit b in blue). **(D–F)** Heat maps (top) and normalized MFR (bottom) across all recorded LS units during the three stage of STFP. **(G)** Scatter plots of normalized MFR across all recorded LS units during cued and uncued eating. Each data point represents a single unit. **(H)** Cumulative probability distributions of the normalized MFR during cued and uncued eating. ^**^P = 0.0054, Kolmogorov-Smirnov test, *z* = 0.247. **(I)** Comparison of normalized MFR across all units during cued and uncued eating. ^**^P = 0.0017, Wilcoxon matched-pairs signed rank test, *W* = –1,619, *r*_rb_ = 0.37, 95% CI [–0.17, –0.0016]. *n* = 93 units from 7 mice. Underlying data can be found in S1 Data.
(TIF)

S6 Fig LFP oscillations in the LS are not significantly different during versus uncued eating. **(A–C)** The normalized power of theta oscillations in the LS remain unchanged during cued and uncued eating. *n.s.*, *P* = 0.36, unpaired *t* test, *t*_(14)_ = 0.94, Cohen’s *d* = 0.47, 95% CI [–0.27, 0.10]. **(D–F)** The normalized power of beta oscillations in the LS remain unchanged during cued and uncued eating. *n.s.*, *P* = 0.37, unpaired *t* test, *t*_(14)_ = 0.92, Cohen’s *d* = 0.46, 95% CI [–0.21, 0.085]. **(G–I)** The normalized power of low gamma oscillations in the LS remain unchanged during cued and uncued eating. *n.s.*, *P* = 0.22, unpaired *t* test, *t*_(14)_ = 1.29, Cohen’s *d* = 0.64, 95% CI [–0.12, 0.030]. **(J–L)** The normalized power of high gamma oscillations in the LS remains unchanged during cued and uncued eating. *n.s.*, *P* = 0.80, Mann Whitney test, *U* = 29, *r*_rb_ = 0.094, 95% CI [–0.12, 0.094]. Underlying data can be found in S1 Data.
(TIF)

S7 Fig Chemogenetic inactivation or activation of LS neurons exerts no effect on total food consumption. **(A)** The total food consumption of two food pairs during hM4Di-mediated inactivation of LS neurons. Cin versus Coc: *n.s.*, *P* = 0.86, Mann Whitney test, *U* = 156, *r*_rb_ = 0.037, 95% CI [–0.051, 0.30], *n* = 18 mice for each group; Cum versus Thy: *n.s.*, *P* = 0.34, unpaired *t* test, *t*_(38)_ = 0.96, Cohen’s *d* = 0.30, 95% CI [0.015, 0.32]; *n* = 18 mice for hM4Di+saline group, *n* = 22 mice for hM4Di+CNO group. **(B)** The total food consumption of two food pairs during hM3Dq-mediated activation of LS neurons. Cin versus Coc: *n.s.*, *P* = 0.96, unpaired *t* test, *t*_(25)_ = 0.06, Cohen’s *d* = 0.022, 95% CI [–0.29, 0.28], *n* = 17 mice for hM3Dq + saline group, *n* = 10 mice for hM3Dq + CNO group; Cum versus Thy: *n.s.*, *P* = 0.18, Mann Whitney test, *U* = 105, *r*_rb_ = 0.27, 95% CI [–0.24, 0.041]; *n* = 17 mice for each group. Underlying data can be found in S1 Data.
(TIF)

S8 Fig Photogenetic inhibition of LS neurons impairs the STFP without influencing sociability. **(A)** Left: schematic of the viral infection of LS neurons in C57BL/6J mice. Right: representative images showing GtACR1-EGFP expression and the fiber placement within the LS. Scale bar = 100 μm. **(B)** Light illumination inhibited current-evoked action potentials in an LS neuron expressing GtACR1. **(C)** Optogenetic inhibition of LS neurons impaired the preference for the cued cinnamon during the STFP. Left: food consumption, EGFP: ^**^P = 0.0090, paired *t* test, *t*_(6)_ = 3.8, Cohen’s *d* = –1.44, 95% CI [–0.13, –0.027]; GtACR1: *n.s.*, *P* = 0.22, Wilcoxon matched-pairs signed rank test, *W* = 16, *r*_rb_ = 0.57, 95% CI [–0.12, 0.58]. Right: preference index, ^*^P = 0.044, unpaired *t* test, *t*_(12)_ = 2.25, Cohen’s *d* = 1.20, 95% CI [–99.27, –1.57]. *n* = 7 mice for each group. **(D)** Optogenetic inhibition of LS neurons impaired the preference for the cued cumin during the STFP. Left: food consumption, EGFP: ^*^P = 0.023, Wilcoxon matched-pairs signed rank test, *W* = −32, *r*_rb_ = –0.89, 95% CI [–0.44, 0.09]; GtACR1: *n.s.*, *P* = 0.64, Wilcoxon matched-pairs signed rank test, *W* = 8, *r*_rb_ = 0.22, 95% CI [–0.33, 0.65]. Right: preference index, *n.s.*, *P* = 0.19, Mann-Whitney test, *U* = 19, *r*_rb_ = 0.41, 95% CI [–107.5, 13.59]. *n* = 8 mice for each group. **(E)** Schematic illustration of the sociability test. **(F, G)** Sociability assessment after optogenetic inhibition of LS neurons. G: interaction time, EGFP: ^**^P = 0.0078, Wilcoxon matched-pairs signed rank test, W = 36, *r*_rb_ = 1, 95% CI [38, 170]; GtACR1: ^**^P = 0.0044, paired *t* test, *t*_(7)_ = 4.13, Cohen’s *d* = 1.46, 95% CI [32.67, 120.1]. Right: discrimination index, *n.s.*, *P* = 0.96, Mann Whitney test, *U* = 31, *r*_rb_ = 0.031, 95% CI [–17.77, 21.66]. *n* = 8 mice for each group. **(H)** Schematic illustration of the social novelty test. **(I, J)** Social novelty assessment after optogenetic inhibition of LS neurons. Interaction time, EGFP: ^*^P = 0.029, paired *t* test, *t*_(7)_ = 2.75, Cohen’s *d* = 0.97, 95% CI [5.45, 72.81]; GtACR1: ^**^P = 0.0078, Wilcoxon matched-pairs signed rank test, W = 36, *r*_rb_ = 1, 95% CI [7, 180]. Discrimination index, *n.s.*, *P* = 0.69, paired *t* test, *t*_(14)_ = 0.40, Cohen’s *d* = 0.20, 95% CI [–39.25, 26.83]. *n* = 8 mice for each group. Underlying data can be found in S1 Data.
(TIF)

S9 Fig The calcium activity of vLS neurons does not discriminate between cued and uncued eating. **(A)** AAV-hSyn-GCaMP6s was injected into the vLS of C57BL/6J mice for calcium signal recording. **(B)** Representative images showing GCaMP6s expression and the fiber placement within the vLS. Scale bar = 50 μm. **(C)** Behavioral assessments of mice during calcium activity recordings. Cued versus uncued: ^**^P = 0.0098, Wilcoxon matched-pairs signed rank test, W = –49, *r*_rb_ = –0.89, 95% CI [–0.26, –0.006]. **(D)** Heat maps (top) and averaged traces (bottom) illustrating the calcium responses evoked in the three sessions. **(E)** Quantification of the AUC of the calcium signal before and after social interaction. *n.s.*, *P* = 0.16, paired *t* test, *t*_(9)_ = 1.53, Cohen’s *d* = –0.48, 95% CI [–83.74, 16.13]. **(F)** Quantification of AUC of the calcium signal during cued and uncued food eating. *n.s.*, *P* = 0.38, paired *t* test, *t*_(9)_ = 0.93, Cohen’s *d* = –0.29, 95% CI [–84.24, 35.12]. **(G, H)** The mean and peak calcium response of LS neurons during cued and uncued food eating. Peak ΔF/F, *n.s.*, *P* = 0.11, Wilcoxon matched-pairs signed rank test, *W* = –33, *r*_rb_ = –0.6, 95% CI [–0.51, –0.016]; mean ΔF/F, *n.s.*, *P* = 0.63, Wilcoxon matched-pairs signed rank test, *W* = –11, *r*_rb_ = –0.2, 95% CI [–0.23, –0.082]. *n* = 10 mice for each group. Underlying data can be found in S1 Data.
(TIF)

S10 Fig Administration of V1aR antagonist (V1aR a) did not affect the preference for the cued cinnamon during the STFP. **(A)** Administration of V1aR a did not affect the preference for the cued cinnamon during the STFP. Food consumption: ACSF: ^*^P = 0.016, Wilcoxon matched-pairs signed rank test, *W* = –34, *r*_rb_ = –0.94, 95% CI [–0.46, –0.019]; V1aR a: ^*^P = 0.025, paired *t* test, *t*_(7)_ = 2.85, Cohen’s *d* = –1.01, 95% CI [–0.30, –0.027]. Preference index: *n.s.*, *P* = 0.22, Mann-Whitney test, *U* = 20, *r*_rb_ = 0.38, 95% CI [–67.31, 7.09]. **(B)** Administration of V1aR did not affect the preference for the cued cumin during the STFP. Food consumption: ACSF: ^**^P = 0.036, paired *t* test, *t*_(7)_ = 2.58, Cohen’s *d* = –0.91, 95% CI [–0.23, –0.010]; V1aR a: ^*^P = 0.013, paired *t* test, *t*_(7)_ = 3.30, Cohen’s *d* = 1.17, 95% CI [–0.24, –0.040]. Preference index: *n.s., P* = 0.33, Mann-Whitney test, *U* = 22, *r*_rb_ = 0.31, 95% CI [–25.33, 47.92]. *n* = 8 mice for each group. Underlying data can be found in S1 Data.
(TIF)

S11 Fig Sex differences in OXTR expression in the dLS. **(A)** Representative images showing OXTR expression in the dLS of male and female mice. Scale bar = 20 μm. **(B)** Quantitative analysis of OXTR mean intensity in the dLS between male and female mice. ^*^P = 0.012, unpaired *t* test, *t*_(23)_ = 2.71, Cohen’s d = 1.09, 95% CI [0.15, 1.08]. n = 15 or 11 randomly chosen fields from 6–8 slices of four mice per groups. **(C)** Quantitative analysis of total OXTR intensity of OXTR^+^ cells in the dLS between male and female mice. ^****^P < 0.0001, Mann-Whitney test, *U* = 913, *r*_rb_ = 0.31, 95% CI [198.9, 500.4]. *n* = 59 or 58 randomly chosen OXTR^+^ cells from 6–8 slices of four mice for the male and female group respectively. Underlying data can be found in S1 Data.
(TIF)

S12 Fig dLS OXTR^+^ neurons show no differential calcium response to either food during innate food preference test. **(A)** Heat maps (top) and averaged traces (bottom) illustrating the calcium responses of the dLS OXTR^+^ neurons during innate food preference test. **(B)** Behavioral assessments of mice during calcium activity recordings. ^**^P = 0.0027, paired *t* test, *t*_(7)_ = 4.52, Cohen’s *d* = –1.60, 95% CI [–0.25, –0.080]. **(C)** Quantification of the AUC of the calcium signal during food eating of innate preference test. *n.s.*, *P* = 0.84, paired *t* test, *t*_(7)_ = 0.21, Cohen’s *d* = –0.073, 95% CI [–251.7, 299.6]. **(D–E)** The mean and peak calcium response of LS neurons during food eating of innate preference test. Peak ΔF/F: *n.s.*, *P* = 0.95, Wilcoxon matched-pairs signed rank test, *W* = –2, *r*_rb_ = –0.056, 95% CI [–0.80, 1.73]; Mean ΔF/F: *n.s.*, *P* = 0.84, paired *t* test, *t*_(7)_ = 0.21, Cohen’s *d* = –0.073, 95% CI [–0.50, 0.60]. *n* = 8 mice for each group. Underlying data can be found in S1 Data.
(TIF)

S13 Fig PLC inhibitor U73122 blocks the excitatory effect of oxytocin on dLS neurons. **(A)** Schematic of the experimental paradigm of bath application with U73122 + TGOT in dLS neurons. **(B–D)** Spontaneous firing activity of dLS neurons during bath application with U73122 or U73122 + TGOT: representative action potential trace (B), raster plots and heatmaps (C), and averaged firing rate across all cells (D). **(E)** Comparison of firing rate under ACSF and U73122 conditions across all cells recorded. *n.s.*, *P* = 0.85, paired *t* test, *t*_(8)_ = 0.19, Cohen’s *d* = –0.065, 95% CI [–0.26, 0.22]. *n* = 9 cells from 5 mice. **(F)** Schematic of the experimental paradigm of bath application with XE991 + TGOT in LS neurons. **(G–I)** Spontaneous firing activity of dLS neurons during bath application with XE991 or XE991 + TGOT: representative action potential trace (G), raster plots and heatmaps (H) and averaged firing rate across all cells (I). **(J)** Comparison of firing rate under ACSF and XE991 conditions across all cells recorded. ^*^P = 0.015, paired *t* test, *t*_(18)_ = 2.68, Cohen’s *d* = 0.61, 95% CI [0.14, 1.03]. *n* = 19 cells from 5 mice. Underlying data can be found in S1 Data.
(TIF)

S1 Data Source data for main figures and supporting figures. (XLSX)

## Abbreviations

ACSF: artificial cerebrospinal fluid
AUC: area under the curve
CNO: clozapine N-oxide
dLS: dorsal LS
IACUC: Institutional Animal Care and Use Committee
LFP: local field potential
LHA: lateral hypothalamic area
LS: lateral septum
mEPSC: miniature excitatory postsynaptic currents
MFR: mean firing rate
mIPSC: miniature inhibitory postsynaptic currents
PCA: principal-component analysis
PLC: phospholipase C
SEM: standard error of the mean
STFP: social transmission of food preference
vLS: ventral LS.
